# Comparative analysis of somatic variant calling on matched FF and FFPE WGS samples

**DOI:** 10.1186/s12920-020-00746-5

**Published:** 2020-07-06

**Authors:** Louise de Schaetzen van Brienen, Maarten Larmuseau, Kim Van der Eecken, Frederic De Ryck, Pauline Robbe, Anna Schuh, Jan Fostier, Piet Ost, Kathleen Marchal

**Affiliations:** 1 Department of Plant Biotechnology and Bioinformatics, Department of Information Technology, IDLab, imec, iGent Toren, Ghent, Belgium; 2 Department of Human Structure and Repair, Ghent University Hospital, Ghent, Belgium; 3 Department of Vascular Surgery, Ghent University Hospital, Ghent, Belgium; 4 Oxford National Institute of Health Research (NIHR) Biomedical Research Centre, University of Oxford, Oxford, United Kingdom; 5 Division of Genomic Medicine, RIKEN Center for Integrative Medical Sciences, Yokohama, Japan; 6 Department of Radiotherapy, Ghent University Hospital, Ghent, Belgium; 7 Department of Genetics, University of Pretoria, Pretoria, SA South Africa

**Keywords:** Somatic variants, FFPE, WGS, Precision oncology, Cohort studies

## Abstract

**Background:**

Research grade Fresh Frozen (FF) DNA material is not yet routinely collected in clinical practice. Many hospitals, however, collect and store Formalin Fixed Paraffin Embedded (FFPE) tumor samples. Consequently, the sample size of whole genome cancer cohort studies could be increased tremendously by including FFPE samples, although the presence of artefacts might obfuscate the variant calling. To assess whether FFPE material can be used for cohort studies, we performed an in-depth comparison of somatic SNVs called on matching FF and FFPE Whole Genome Sequence (WGS) samples extracted from the same tumor.

**Methods:**

Four variant callers (i.e. Strelka2, Mutect2, VarScan2 and Shimmer) were used to call somatic variants on matching FF and FFPE WGS samples from a metastatic prostate tumor. Using the variants identified by these callers, we developed a heuristic to maximize the overlap between the FF and its FFPE counterpart in terms of sensitivity and precision. The proposed variant calling approach was then validated on nine matched primary samples*. Finally, we assessed what fraction of the discrepancy could be attributed to intra-tumor heterogeneity (ITH), by comparing the overlap in clonal and subclonal somatic variants.*

**Results:**

We first compared variants between an FF and an FFPE sample from a metastatic prostate tumor, showing that on average 50% of the calls in the FF are recovered in the FFPE sample, with notable differences between callers. Combining the variants of the different callers using a simple heuristic, increases both the precision and the sensitivity of the variant calling. Validating the heuristic on nine additional matched FF-FFPE samples, resulted in an average F1-score of 0.58 and an outperformance of any of the individual callers. In addition, we could show that part of the discrepancy between the FF and the FFPE samples can be attributed to ITH.

**Conclusion:**

This study illustrates that when using the correct variant calling strategy, the majority of clonal SNVs can be recovered in an FFPE sample with high precision and sensitivity. These results suggest that somatic variants derived from WGS of FFPE material can be used in cohort studies.

## Background

Cohort analysis in which comprehensive genomic data of large patients’ cohorts are being coupled with clinical information offers a vast potential for precision oncology. So far, most large cohort studies relied on whole exome sequencing (WES) or WGS of FF tumor material. However, the preservation of and access to FF tissues can be limited. Indeed, in routine clinical practice, FF samples are rarely available due to logistic reasons: they are difficult to collect, prepare and are expensive to store. Optimally exploiting available patients’ cohorts would therefore require collecting sequence information from FFPE samples that are collected in routine standard of care for histopathological diagnosis. This poses a problem as DNA extracted from FFPE specimens presents degradation such as nucleic acid fragmentation, DNA crosslinks, abasic sites leading to localized DNA denaturation, strand breaks, and deamination leading to C > T mutations [1–3].

Several studies have established that sufficiently high-quality DNA can be derived from FFPE material. Although the processing and storage affect the quality of the DNA and subsequent next generation sequencing (NGS) data [4–7], for most samples, enough qualitative DNA can be collected to perform NGS assays in order to identify copy number variations (CNVs) and single nucleotide variations (SNVs) [8–10]. Most studies that compared somatic variants, obtained from sequencing matched FF and FFPE samples, are based on whole exome sequencing (WES). Depending on the study [11–13], an overlap between 54 and 90% was found in somatic variants obtained from matched FF and FFPE samples. Differences in results can be attributed to differences in studied cancer types (which might differ in ITH) and the fact that different variant callers and quality thresholds were used.

In this work, we performed an in-depth comparison of the degree to which the same somatic SNVs can be called using WGS of FF and FFPE samples extracted from the same tumor. This has been done on samples from a metastatic prostate tumor from the Ghent University Hospital (UZ). Based on these findings, we developed a variant calling strategy that uses an ensemble of different variant callers. The proposed variant calling approach is then validated on nine primary samples from Robbe et al. [14] and although these samples were collected with a slightly different aim and using different protocols, our approach robustly identified true positive variants in the FFPE samples. Finally, we demonstrated that variants that are not common to both the FF and the FFPE samples are mainly subclonal, such that the discrepancy in variant calls observed between the FF and the FFPE samples might be attributed to ITH. Our results complement the findings of Robbe et al. [14] and demonstrate the feasibility of accurately detecting clonal variants in FFPE samples. However, in contrast to previous studies, that focused mainly on maximizing the specificity, our variant calling strategy results in both a high precision and sensitivity. This suggests that FFPE samples can be used for cohort analyses in cancer research e.g. for driver mutation identification.

## Methods

### Patient and samples

For this study, a patient from Ghent University Hospital (UZ001) with isolated pulmonary recurrence of prostate cancer after initial definitive local therapy was selected for who we had both an FF and an FFPE sample from the solitary pulmonary metastasis. The prostatic origin of the lung metastatic adenocarcinoma was confirmed by pathological review (J.V.D., S.V. and K.V.D.E.). Microscopically, the pulmonary metastasis was composed of eosinophilic tumor cells with very large pleomorphic hyperchromatic nuclei and prominent nucleoli, which exhibited a cribriform pattern (see Suppl. Fig. 1 A, Additional File 2) with negative staining for CK7 and TTF-1 and positive PSA staining (see Suppl. Fig. 1 B, C and D, Additional File 2); these findings were compatible with metastatic prostate cancer. Two samples from this pulmonary metastasis had been obtained, one had been stored as FFPE and one as FF. Whole blood was collected and informed consent was obtained at time of clinical follow-up.

### Pathologic quality control (QC) of UZ001 samples

For both the FF and the FFPE samples of patient UZ001, 5 μm-thick haematoxylin and eosin-stained slides were prepared and independently evaluated by two genitourinary pathologists (J.V.D. and S.V.) to determine the tumor cellularity. For the FFPE tissue, 11 adjacent 5 μm-tick sections were prepared. The first 10 sections were used for DNA extraction, whilst the last section served as reference to indicate a tumor-rich area suitable for macro-dissection (more than 70% tumor cellularity). Manual macro-dissection was performed using sterile scalpel blades. Information about input materials is displayed in Table 1.

**Table 1:** Input material (in ng) per sample analyzed of patient UZ001

Sample type	FFPE	Blood	FF
**Input (ng)**	300	300	500

### Preparation steps and sequencing of UZ001 samples

The genomic DNA (gDNA) was extracted from the FFPE tissue using the proprietary method of Wuxi (NextCODE SeqPlus extraction protocol) and from the FF tissue with QIAamp DNA Mini Kit (Qiagen) according to the manufacturer’s instructions. gDNA was extracted from a 200 μL EDTA-whole blood sample using the QIAamp® Blood Mini Kit (Qiagen) with QIAcube according to the manufacturer’s instructions. The DNA samples were quantified with a Qubit 3.0 fluorescence spectrometer (Life Technologies, Waltham, MA USA) using a Qubit dsDNA BR assay kit. Covaris has been used for DNA shearing. TruSeq® Nano DNA Library Prep (Illumina) has been used for library construction. The Illumina sequencing platform HiSeqX PE150 has been used for WGS. The mean coverage was of 30X for the blood sample and 100X for the tumor samples.

### Samples used for validation

Access to tumor and matched control samples from a pilot study of the 100,000 Genomes Project England has been obtained. For nine patients, an FF and an FFPE sample from the same tumor (four prostate and five renal tumors) and a matched blood sample (control) were available. All the FFPE samples from prostate tumors were prepared with the same protocol (temperature, time and preparation kit). The protocol was the same for renal tumors except that another preparation kit was used for some FFPE samples (see Table 2). More details about the preparation steps and sequencing are available in the paper of Robbe et al. (2018).

**Table 2:** Description of samples available from a pilot study of the 100,000 Genomes Project England

Patient ID	FF sample ID	FFPE sample ID	Blood sample ID	Cancer Type	FFPE Prep Kit
**GeL007**	LP2000456-DNA_A01	LP2000467-DNA_A01	LP2000446-DNA_A01	Prostate	Covaris
**GeL008**	LP2000457-DNA_A01	LP2000468-DNA_A01	LP2000447-DNA_A01	Prostate	Covaris
**GeL024**	LP2000558-DNA_A01	LP2000596-DNA_A01	LP2000577-DNA_A01	Prostate	Covaris
**GeL028**	LP2000559-DNA_A01	LP2000597-DNA_A01	LP2000578-DNA_A01	Prostate	Covaris
**GeL004**	LP2000462-DNA_A01	LP2000691-DNA_A01	LP2000452-DNA_A01	Renal	Qiagen
**GeL032**	LP2000460-DNA_A01	LP2000621-DNA_A01	LP2000450-DNA_A01	Renal	Covaris
**GeL065**	LP2000498-DNA_A01	LP2000645-DNA_A01	LP2000484-DNA_A01	Renal	Covaris
**GeL300**	LP2000696-DNA_A01	LP2000683-DNA_A01	LP2000695-DNA_A01	Renal	Qiagen
**GeL365**	LP2100046-DNA_A01	LP2000888-DNA_A01	LP2000888-DNA_C03	Renal	Covaris

### Somatic variant calling

Quality checking were obtained by running GATK Picard tools (https://broadinstitute.github.io/picard/). For somatic variant calling, we used Strelka2 from Illumina [15], Mutect2 from GATK (version 4.1.2) [16], and Shimmer (version 0.2) [17] with default settings. VarScan2 (version 2.2.3) [18] was run without imposing a minimal Variant Allele Frequency (VAF) threshold. To select the most reliable somatic calls, *FilterMutectCalls* with default parameters was applied on Mutect2, *somaticFilter* to the VarScan2 output without imposing the default threshold of minimal VAF and no additional somatic filters were applied to Strelka2 and Shimmer output. Table 3 provides a summary of the main parameters of the four variant callers used. The same variant calling settings were used to call somatic variants on all samples analyzed in this study.

**Table 3:** Summary of the main parameters used for Strelka2, Mutect2, VarScan2 and Shimmer

Strelka2	Shimmer
Min Somatic EVS = 7	Max q-value acceptable FDR = 0.05
**Mutect2**	**VarScan2**
Min base quality score = 10 Min Phred-scaled confidence threshold = 10 Min TLOD = 5.3 Min NLOD = 2.3 Sample ploidy = 2 *FilterMutectCalls:* Min MedianBaseQuality = 20 Min MedianMappingQuality = 30	Min coverage in normal, in tumor = 8, 6 Min variant allele frequency = 0.01 Max somatic *p*-value = 0.05 *somaticFilter:* Min variant allele frequency = 0 Min read depth = 10 Min average quality = 20 Max somatic p-value = 0.01

The default settings used by VarScan2 impose a threshold for the minimum VAF at 0.2 (see *somaticFilter* in VarScan2’s Online Manual). This prevents VarScan2 from detecting the variants with low allele frequency in the FFPE sample. To recover also these variants and hence maximize the overlap with the other callers, we ran VarScan2 without the constraint on the minimal VAF threshold. Using this non-default setting resulted in VarScan2 detecting more variants with VAF lower than 0.2 and on overall increased the overlap in somatic variants detected by Strelka2, Mutect2 and Shimmer on the same sample while also decreasing significantly the number of variants uniquely called by VarScan2 (data not shown). Although we expected intuitively that most calls obtained by VarScan2 without the VAF constraints would also be present in calls from VarScan2 with default parameters, this appeared not to be the case (and we could not find any reasonable explanation for this).

#### Identifying the stringency of the calls

For Strelka2, the stringency of the calls was determined by the *Somatic EVS*, for Mutect2 by the *TLOD* scores, for VarScan2 by the *somatic p-values* and for Shimmer by *q-values*. The higher the scores were for Strelka2 and Mutect2, the more significant were the variants. For VarScan2 and Shimmer, the smaller values were the most significant.

#### Measure to evaluate the concordance between FF and FFPE samples

The overlap between reported calls in matching FF and FFPE samples is reported in terms of sensitivity and precision using the variants obtained in the FF sample as gold standard. A somatic variant was considered present in both samples if in both samples the variant was located at an identical chromosomal position, and if the reference and the alternative alleles were identical.

#### Identifying the threshold maximizing the overlap between FF and FFPE samples

For each variant caller, we searched for an optimal significance threshold in the FFPE sample to obtain the largest concordance between samples (maximal F1-score). The screening space for the optimization of the significance threshold is displayed in Table 4.

**Table 4:** Screening space for the threshold optimization for each variant caller

Strelka2	Shimmer
Somatic EVS from 5 to 20 (steps of 0.25)	Q-value from 0.0005 to 0.05 (steps of 0.0005)
**Mutect2**	**VarScan2**
TLOD from 0 to 200 (steps of 1)	Somatic p-value from 0.00005 to 0.01 (steps of 0.00005)

### Mutational profile analysis

The mutation profile of a sample is created by measuring the frequency of SNVs in each of the 96 mutation types. Those 96 mutation types represent every possible single base substitution (C > A, C > G, C > T, T > A, T > C, T > G) and the 5′ and 3′ surrounding nucleotides, also called trinucleotide context (e.g. A [C > A] T, A [C > A]C). The mutational profiles were used at first to confirm that the same mutational patterns were observed between the matching FF and FFPE samples. Hereto, we measured for each caller the cosine similarity between the mutational profiles of the matching FF and FFPE samples. A higher cosine similarity between the FF and the FFPE mutational profiles indicates that the variant caller returns the same mutation types in both samples and hence that no FFPE specific mutational patterns were present that could reflect FFPE related biases.

In addition, the mutational profiles were used to assess whether the FFPE sample was biased towards specific sequencing artefacts reflected by known COSMIC signatures. Hereto, the *fit_to_signatures* function from Mutational Patterns R package [19] was used to decompose mutational profiles into pre-defined COSMIC single base substitution signatures [20]. This function searches for the optimal linear combination of COSMIC signatures that most closely reconstructs the mutation profile of the sample by solving a non-negative least-squares problem, where every COSMIC signature is defined by a unique combination of the 96 mutation types. Subsequently, we assessed for each sample to what extent COSMIC signatures representative for sequencing artefacts contributed to the most optimal reconstruction of its mutational profile. Signatures reported in COSMIC as potentially representative for artefacts but that were found in multiple patients with prostate and renal cancer in COSMIC (bold and underlined in Suppl. Table 1**,** Additional File 1) were not considered as representative for artefacts in the analysis of the samples that originated from respectively a prostate or renal tumors. Only samples that displayed a minimal cosine similarity of 0.9 between the reconstructed mutation profile of COSMIC signatures and the original mutation profile were used to estimate the contribution of sequencing artefact to the mutation profiles.

### Copy number variation calling

Copy Number Variant (CNV) calling was done using the GATK (version 4.1.2) somatic CNV calling pipeline. Read counts per 1000 bp intervals were corrected for GC bias and the samples from a pilot study of the 100,000 Genomes Project England were denoised using a panel of normals (PoNs). We noted that these correction steps resulted in less noisy count profiles (see Suppl. Fig. 2**,** Additional File 2), but that in almost all samples, the final FFPE CN segments were more fragmented compared to those in the FF sample (see Suppl. Fig. 3**,** Additional File 2). To assess to what extent these scattered fragments resulted in a different CN status, we calculated the overlap of the segments, see Suppl. Table 2**,** Additional File 1. From this table, it can be seen that the fragmented segments in the FFPE sample often resulted in false positive amplifications and, to a lesser extent, deletions. Since the VAF of variants in those segments would be corrected in the FFPE sample and not in the FF sample, the CN calling could introduce an additional bias in the analysis of VAFs. Therefore, we only selected variants called by at least two callers in segments that are diploid in both the FF and the FFPE samples, referred to as bona fide *diploid variants*. For the same reason, variants on the sex chromosomes were excluded from the analysis. Suppl. Table 3 (Additional File 1) shows how many variants were retained for each sample.

### Purity correction

To assess the purity of the samples, we could rely on the pathologists’ estimate, where samples with a purity below 40% were discarded. To compensate for potential bias in the pathologist’s estimate, we also applied two different tools to estimate tumor purity. TPES [21] estimates tumor content based on the set of variants called by at least two variant callers in each sample and was complemented with the FACETS tool [22], which looks at common human SNP sites and measures purity based on an Expectation Maximization of the ASCAT formulas [23]. For TPES, the variants obtained through our variant calling strategy were used as inputs (VCFs), together with the *seg* files obtained from the GATK CNV pipeline. Because those filtered VCFs contain less, but high-confident variants, we had to lower the *minSNVs* parameter to 3 [23]. FACETS was run using default parameters [22]. For patient UZ001, matching RNA material was available such that we could assess Tumor Infiltrating Leukocytes (specifically we tested for infiltration of B cells, CD4 T cells, CD8 T cells, Monocytes, Neutrophils and NK cells) through transcriptome deconvolution, done using EPIC [24].

### Clonal variant extraction

To identify clonal variants, we grouped the bona fide diploid variants into different categories using the same approach as the TPES tool [21]. First, a kernel distribution is estimated for the VAF distribution, where a Gaussian kernel was used [25]. The optimal bandwidth for the kernel function was determined using cross-validation over a grid [0.1, 0.01], optimizing the log likelihood of the data [25]. Once the kernel density was estimated, the VAF data was cut into segments at each local minimum of the density distribution (see Suppl. Fig. 4, Additional File 2, for an illustration). Then, by visual inspection of the plots, the clonal population for each sample was determined. All high-confidence variants with a VAF lower than the variants in the clonal subpopulation were labelled subclonal.

## Results

### In depth analysis of variant calling on matching FF and FFPE metastatic prostate tumor samples (UZ001)

We aimed at testing to what extent WGS of FF- and FFPE-derived material results in the identification of the same somatic SNVs. Hereto, we used one FF and one FFPE sample of the same metastatic prostate tumor from patient UZ001 (see **Methods**). We opted for a metastatic sample as this is in general more homogenous than primary samples, reducing differences in variant calls due to ITH and sampling variation. A matching blood sample, taken from the same patient, was used as a reference to identify germline mutations.

Quality checking showed that in the matching FF and FFPE samples, a comparable number of variants was called (see Suppl. Tables 4 and 5**,** Additional File 1). In addition, no bias towards FFPE specific C > T deamination artefacts was observed (see Suppl. Table 4**,** Additional File 1). Both samples showed a similar average coverage (see Suppl. Table 5, Additional File 1). To ensure that comparing the somatic variants called from the matching FF and FFPE samples would be independent of the specificities of the variant caller, we used four somatic variant callers, i.e. Strelka2 [15], Mutect2 [16], VarScan2 [18] and Shimmer [17].

### Comparison of variants detected in respectively the FF and FFPE sample

At first, we compared the extent to which each caller identifies the same variants in the matching FF and FFPE samples. All variant callers except VarScan2 were run under default settings (see **Methods**). In general, more calls were reported in the FFPE than in the FF sample, except for VarScan2 (see Suppl. Fig. 5, Additional File 2). Table 5 shows the overlap between somatic calls in the matching FF and FFPE samples in terms of sensitivity and precision using the calls from the same variant caller on the FF sample as gold standard. Overall, variant callers have an average sensitivity and precision of respectively 50.97 and 52.41%, meaning that 50.97% of the variants from the FF sample are also detected in the FFPE sample and 52.41% of the FFPE somatic variants are detected in the FF sample. We also report the F1-score, which is the harmonic mean of sensitivity and precision. On average, the variant callers achieved an F1-score of 50.05%. Among the four variant callers considered in this study, Strelka2 achieved the highest F1-score.

**Table 5:** Performance measures of calls considering the FF sample as gold standard for each variant caller (UZ001)

Variant caller	FF	FFPE	Overlap	Sensitivity	Precision	F1-score
**Strelka2**	6292	6761	4225	0.6715	0.6249	0.6474
**Mutect2**	10,460	11,815	5755	0.5502	0.4871	0.5167
**VarScan2**	4067	1760	883	0.2171	0.5017	0.3031
**Shimmer**	8109	10,080	4865	0.6000	0.4826	0.5349

To get an idea of how good the sensitivity and precision from Table 5 are, we compared the calls from different variant callers on the same sample. In general, the difference between variant callers on the same sample is larger than the difference between the FF and the FFPE sample observed for the same caller (see Suppl. Fig. 5 and 6, Additional File 2). This implies that the choice of variant caller is at least as important as whether or not the sample was FF or FFPE. Overall, the overlap between variant callers on the same sample is low, especially for the FFPE sample (see Suppl. Fig. 6, Additional File 2). Shimmer agrees the least with the other variant callers, whereas Strelka2 and Mutect2 tend to mutually agree in both the FF and the FFPE samples (see Suppl. Tables 6 and 7**,** Additional File 1).

To further confirm that no bias towards specific mutational patterns was present between the FF and the FFPE sample that could indicate FFPE related artefacts, we assessed, for each caller, the cosine similarity between the mutational profiles of the FF and the FFPE sample (see **Methods**). Suppl. Table 8 (Additional File 1) illustrates that the mutational profiles of variants reported by each caller were consistent between the FF and the FFPE sample except for Shimmer, indicating the absence of any bias towards specific mutational patterns in the FFPE sample.

While the choice of variant caller turns out to play a pivotal role in the calls that are obtained, the basic analysis above does not account for the properties of the called variants. Indeed, we will demonstrate that the variants considered in Table 5 represent both true variants and artefacts of the used variant caller, consistently made in the FF and the FFPE sample. To assess the relevance of the calls made by each of the callers in the overlap between the FF and the FFPE sample, we first assessed to what extent each of the callers tends to reconstruct the same (sub) clonal subpopulations in both the FF and the FFPE samples and secondly whether high confidence calls are consistent between both sample types.

#### Assessing whether variants called in the FF and FFPE samples represent the same (sub) clonal populations

**Fig. 1:**
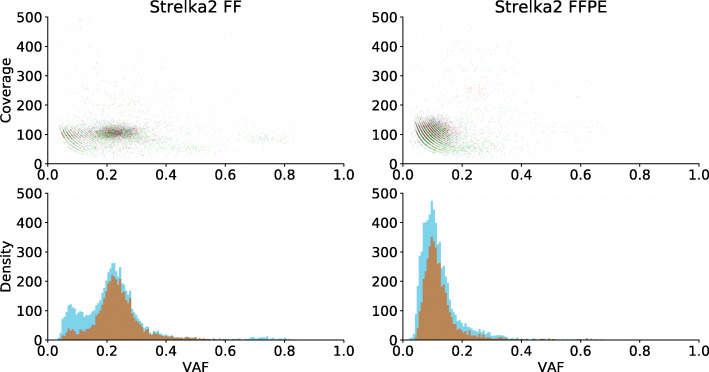
(Sub) clonal populations detection in the FF (left) and the FFPE (right) sample using Strelka2 (UZ001). The upper panel shows coverage as a function of the VAF [26], where a higher variance in the coverage can be observed for the FFPE sample. The lower panel shows the distribution of the VAFs. The blue distribution denotes all calls made in a given sample, while the orange distribution shows only the calls common to the FF and the FFPE sample

Given that the FF and the FFPE samples should have a similar subclonal structure, as they derive from the same metastatic tumor, we judged the relevance of the variants called by either method by assessing whether they corresponded in the FF and the FFPE sample to the same (sub) clonal populations. To visualize the different populations in each sample, we plotted for all detected variants the coverage as a function of the VAF and the corresponding histogram representing the distribution of the VAFs [26]. Figure 1 shows the results for Strelka2 in both the FF and the FFPE samples. Similar figures for the three other variant callers can be found in the **Supplementary Figures,** Additional File 2. Note that the VAF estimate of the major distributions slightly differs between the different tools because of small discrepancies in their definition of the VAF (see Suppl. Fig. 7, Additional File 2).

According to Strelka2 (Fig. 1), two distinct clusters of variants can be observed in the FF sample, denoted by the blue bars in the histogram: a small cluster at a VAF of 0.05 and a larger cluster around a VAF of 0.25. In the FFPE sample, only one cluster can be observed around a VAF of 0.15. The calls that are common to the FF and the FFPE sample are shown in orange, demonstrating that the peak at a VAF of 0.25 in the FF sample does indeed shift to 0.15 in the FFPE sample. Almost all variants from the largest cluster in the FF sample, i.e. at a VAF of 0.25, are identified in the FFPE sample but most of the FF calls belonging to the smaller peak at 0.05 are not detectable in the FFPE sample. This shows that most variants from the major population identified in the FF sample are recovered in the FFPE sample.

We observed similar results for Mutect2 with the peak representing the major cluster at VAF 0.25 in the FF sample being shifted to a lower VAF in the FFPE sample (see Suppl. Fig. 8, Additional File 2). For VarScan2, only a small proportion of the variants detected at an average VAF of 0.25 in the FF is recovered in the FFPE sample (see Suppl. Fig. 9, Additional File 2). Shimmer could barely detect the cluster of variants at VAF 0.25 in the FF and completely misses the major cluster in the FFPE sample (see Suppl. Fig. 10, Additional File 2). A deeper analysis shows that Shimmer reports many somatic variants in the tumor sample that have a VAF above zero in the normal sample (putative germline calls). This behavior is not expected and not observed for any of the other variant callers, explaining the poor overlap between Shimmer and the other variant callers (see Suppl. Tables 6 and 7**,** Additional File 1). Only keeping for Shimmer the variants with zero VAF in the normal sample filtered those putative germline calls and allows to better recover a cluster of variants at VAF 0.25 in the FF, but still not in the FFPE sample (see Suppl. Fig. 11, Additional File 2).

In addition to the cluster at VAF 0.25, VarScan2 also reports a peak of variants at an average VAF of 0.5 in the FF sample. These are likely germline variants as more than 65% of variants with VAF above 0.5 are present in dbSNP database (see Suppl. Table 9**,** Additional File 1). Except for Mutect2, which has a similar number of germline calls above and below a VAF of 0.5, other callers reported up to 8 times more calls from dbSNP at a VAF above 0.5. This suggests that calls reported with VAF above 0.5 are more likely to be false positives. Indeed, most of the calls with VAF above 0.5 detected consistently in both the FF and the FFPE samples tend to be residual germline calls.

Hence, the cluster of variants detected at VAF 0.25 likely represents the major population of variants in the metastatic sample. All variant callers except Shimmer could at least partially recover these variants in the FFPE sample albeit at lower VAF (see Suppl. Fig. 12, Additional File 2). Imposing a zero VAF criterion in the normal sample helped Shimmer to recover the major cluster of variants that was also recovered by the other callers, but only in the FF sample. In addition, only a minority of calls (813) are retained of which most (604) were also reported by other callers. This indicates that many of the calls uniquely made by Shimmer without imposing the zero VAF criteria in the normal sample and reported in Table 5 are likely spurious calls, despite being consistently detected in both the FF and the FFPE samples. For the remainder of the analysis, variants reported by Shimmer with a positive VAF in the normal sample were filtered.

#### Comparing for each of the different callers the significance levels between the FF and FFPE sample

To assess the relevance of the calls in the overlap between the FF and the FFPE sample, we also compared, for each caller, the distributions of the significance scores of the somatic variant calls detected in the FFPE sample that were also called in the FF sample, hereby assuming that the FF sample is less prone to artefacts and constitutes the reference as to what should be detected. Table 6 shows that calls reported in both samples typically received lower significant scores in the FFPE than in the FF sample, which can be explained by the discrepancies in VAF observed in Fig. 1.

**Table 6:** Average significance scores for somatic variants reported in both samples for each variant caller (UZ001). For Strelka2 and Mutect2, a higher Somatic EVS and TLOD means a higher confidence in the calls, while for VarScan2 and Shimmer a lower value implies a higher confidence

Sample	Strelka2	Mutect2	VarScan2	Shimmer
**FF**	17.33	51.88	0.0024	0.0160
**FFPE**	14.34	26.00	0.0038	0.0166

**Fig. 2:**
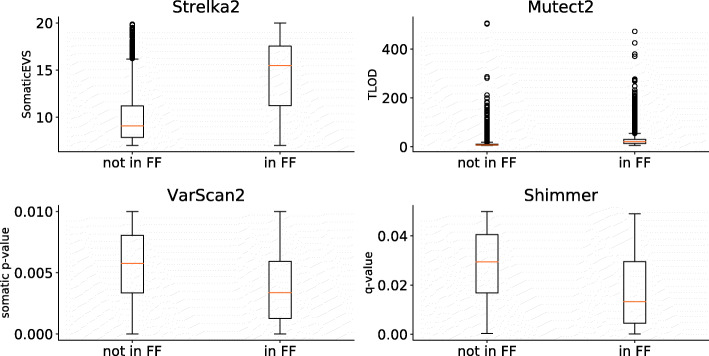
Boxplots comparing the significance level of FFPE reported or not in FF (UZ001). For Strelka2 and Mutect2, a higher Somatic EVS and TLOD means a higher confidence in the calls, while for Varscan2 and Shimmer a lower value implies a higher confidence

In addition, the boxplots in Fig. 2 show that the FFPE calls that were also made in the FF sample, received a relatively higher significance score than FFPE calls not made in the FF sample. This shows that the most reliable variants in the FFPE sample generally correspond to those detected in the FF sample.

For each caller, we calculated the correlation between the significance levels reported in the FF and in the FFPE sample of somatic variants called in both samples. Suppl. Table 10 (Additional File 1) shows that the significance levels of Strelka2 and Mutect2 are significantly correlated in both the FF and the FFPE samples. In addition, the significance levels between the callers themselves seems to be consistent between the FF and the FFPE sample. For Shimmer and VarScan2, this consistency between both samples cannot be observed. Furthermore, all callers assign a similar relative rank to common variants in the FF sample but not necessarily in the FFPE sample (i.e. the same variant is ranked high by all callers in the FF sample but in the FFPE sample the ranks for that variant vary depending on the caller). This indicates that these variant callers are less performant in the FFPE sample. A possible explanation for this could be that the underlying hypergeometric testing procedure (used in both VarScan2 and Shimmer) cannot cope with the lower VAFs present in the FFPE sample.

#### Relation between the (sub) clonal structure and the significance level

**Fig. 3:**
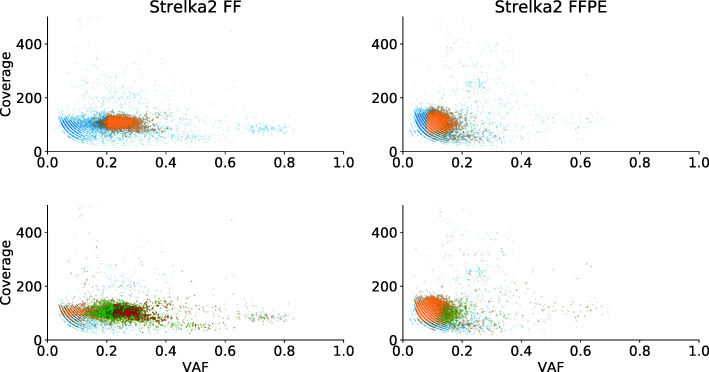
Coverage versus VAF for variants reported by Strelka2, comparing FF (left) against FFPE (right) (UZ001). This plot is identical to the upper panel of Fig. 1 but with a color used to indicate the most significant calls. The upper panel shows the 25% highest confidence calls in orange and the lower confidence in blue. The lower panel shows which calls are also found by other callers where blue = unique calls, orange = calls reported by two callers, green = calls reported by 3 callers, red = calls reported by 4 callers

To investigate the relation between the (sub) clonal structure and the significance of the calls, we map for each variant caller the 25% highest confidence calls on the coverage versus VAF plots. The upper panel of Fig. 3 shows how, for Strelka2, these most significant calls are located around a VAF of 0.25 in the FF and 0.15 in the FFPE sample, and hence make up the aforementioned major subpopulation that was detected in both the FF and the FFPE samples.

For Mutect2, most significant calls also belonged to this cluster of variants (see Suppl. Fig. 13, Additional File 2). For VarScan2 and Shimmer (see Suppl. Fig. 14–16, Additional File 2), a similar effect is observed, although not as pronounced as in Strelka2 and Mutect2. Subsequently, many of the highly significant calls belong to the major subpopulation at a VAF of 0.25 in the FF and 0.15 in the FFPE sample; such that the analysis of the significance levels and the clonal subpopulations points at the existence of a highly confident subset of variants, present in both the FF and the FFPE samples.

In addition, the lower panel in Fig. 3 shows that many variants called by other callers are also located in the variant cluster (see also the lower panels of Suppl. Fig. 13–16, Additional File 2). This is in line with the observation that for each variant caller (except Shimmer), calls made by any of the other three variant callers in general received a higher significance especially in the FF sample (Suppl. Fig. 17 and 18, Additional File 2). Using two criteria, based the (sub) clonal structure (Fig. 1) and significance score of the variants (Fig. 2), we could see that calls common to the FF and the FFPE sample tend to belong to the major subpopulation and are highly significant. Importantly, the lower panel of Fig. 3 and Suppl. Fig. 17 and 18 (Additional File 2) show that variants called by more than one caller tend to satisfy these two criteria as well. In the next section, we investigated how well these calls, reported by more than one variant caller, overlap between the FF and the FFPE sample.

#### Optimizing for each variant caller its significance threshold by optimizing the overlap in variants between the FF and FFPE sample

To assure that only biologically relevant calls are considered, we first identified a highly reliable subset of calls in the FF sample. This subset, referred to as the *ground truth*, can then be used to more qualitatively assess how well each caller can recover these highly reliable calls in the FFPE sample. From our analysis above, an ideal ground truth would consist of all highly reliable calls made in the reference FF sample that also represent the major population in the metastatic sample. We have shown that calls made by at least two callers tend to satisfy these criteria. Therefore, the ground truth is defined as the union of all calls that were detected by at least two callers in the FF sample.

Our previous analysis also shows that the subpopulation represented by the ground truth is also present in the FFPE sample albeit at lower VAF (median VAF of 0.1083 in the FFPE instead of 0.2323 in the FF sample). Hence, fully recovering the subpopulation of highly significant variants from the FFPE sample will require the identification of a significance threshold in the FFPE sample. Because calls in the FF sample were reported with a higher significance scores, the threshold in the FFPE sample will typically be lower than the threshold that would be necessary in the FF sample to capture the ground truth (see Suppl. Fig. 17 and 18, Additional File 2). In addition, previous analysis shows that it is feasible to set a threshold in the FFPE sample as calls common to the FF and the FFPE sample tend to have a higher significance level in the FFPE sample (see Fig. 2). However, the lower VAF in the FFPE sample complicates identifying a threshold that distinguishes the true calls from the noise (the significance distribution of noisy and true calls starts overlapping, see Suppl. Fig. 18, Additional File 2). We determined, for each variant caller, a threshold in the FFPE sample that optimized the F1-score with the FF-derived ground truth (i.e. that optimizes the tradeoff between precision and sensitivity in recovering the ground truth). Table 3 shows for each caller their sensitivity and precision in recovering the ground truth. This table also shows that Strelka2 and Mutect2 are performing the best in recovering from the FFPE sample the calls that belong to the ground truth. Table 7 now quantitatively illustrates how Shimmer underperforms on the ground truth despite calling consistently the same mutations in the FF and the FFPE sample, which was shown in Table 5. This suggests that most of the calls made by Shimmer in the overlap between the FF and the FFPE sample are likely spurious. The same is to some extent true for VarScan2 because of the high number of residual germline calls.

**Table 7:** Optimized F1-scores of calls for each variant caller considering FF sample as gold standard (UZ001). Optimized F1-scores of calls made by each variant caller considering FF sample as gold standard

Caller - threshold	FF (gold std.)	FFPE	Overlap	Sensitivity	Precision	F1-score
**Strelka2–9.5**	4656	4559	3316	0.7122	0.7274	0.7197
**Mutect2–13**	4656	5658	3418	0.7341	0.6041	0.6628
**VarScan2–0.00995**	4656	1755	425	0.1045	0.2422	0.1460
**Shimmer – 0.0495**	4656	262	16	0.0197	0.0611	0.0298

Table 7 gives an estimate of the expected overlap between the FF and the FFPE sample after optimizing the thresholds in the FFPE sample using the ground truth based on the variants detected in the FF sample. However, often only FFPE samples are available, such that the stringency thresholds cannot be optimized based on observations in the FF sample. Therefore, rather than optimizing the threshold for each caller separately, we assessed to what extent combining the output of different variant callers in the FPPE sample allows recovering the ground truth from the FF sample.

Table 8 shows how taking the intersection of the four variant callers maximizes the precision but comes at the expense of losing almost all sensitivity. As discussed above, Shimmer can barely retrieve the cluster representing the major subpopulation in the FFPE sample (see Suppl. Fig. 15 and 16, Additional File 2) and most of the calls retrieved by Shimmer in the FFPE sample are unique (even after correcting for the so-called somatic calls with high VAF in the normal sample). Because the intersection seems too strict and limits the sensitivity, we considered calls reported by at least three callers. It results in a high precision but still relatively low sensitivity in recovering the ground truth (Table 8) because VarScan2 reports less calls in the FFPE than in the FF sample and loses many true positive calls. Using the calls returned by at least two of the four callers in the FFPE sample drastically increases sensitivity while only slightly decreasing precision. The performance here is even better than the performance of the best caller (Strelka2) that was obtained after optimizing its stringency thresholds based on the ground truth. This indicates that there is some complementarity in the calls made by different callers.

**Table 8:** Strategies to retrieve the ground truth calls from the FF in the FFPE sample (UZ001)

Reported by … (in FFPE)	FF (gold std.)	FFPE	Overlap	Sensitivity	Precision	F1-score
**at least 1 caller**	4656	16,020	4155	0.8924	0.2594	0.4019
**at least 2 callers**	4656	4232	3684	0.7912	0.8705	0.8290
**at least 3 callers**	4656	340	325	0.0698	0.9559	0.1301
**all 4 callers**	4656	8	8	0.0017	1	0.0034

Note that when considering only variants called by at least two variant callers in both samples, the cosine similarity between the mutational profiles was of 0.995 which is higher than the cosine similarities between the FF and the FFPE samples for any of the caller (see Suppl. Table 8**,** Additional File 1). This further confirms the consistency between the calls made on the FF and the FFPE samples. Moreover, we decomposed the mutational profiles of the variants called in both the FF and the FFPE samples into COSMIC signatures [20]. Some of those signatures are representative for sequencing artefacts (see Suppl. Table 1**,** Additional File 1). When considering the union of calls made by the four callers in the FF and the FFPE sample, the contribution of the COSMIC signatures representative for sequencing artefacts to the total mutational profile was of 15.21 and 17.68% respectively. When considering only variants called by at least two callers those proportions decreased significantly (8.22% for the FF and 9.36% for the FFPE sample) indicating that considerably less spurious calls related to sequencing artefacts were made.

### Validation of the *at least two* variant calling strategy on samples from the 100,000 genomes project England

#### Comparison of variants detected in respectively the FF and FFPE samples

Nine paired FF-FFPE samples from the same tumors obtained from a pilot study of the 100,000 Genomes Project England have been analyzed in order to validate our approach. As for patient UZ001, variant callers reported systematically more calls in the FFPE than in the FF samples (except for Shimmer in GeL365 patient). Either Strelka2 or Mutect2 achieved the highest F1-scores. Overall, the highest F1-score per variant caller varied between 10.12% (GeL032) to 77.30% (GeL300) with an average of 44.10% (see Table 9). On average, the variant callers were less consistent in the FFPE samples than in the FF samples and the difference between callers in the FFPE sample was larger than the difference between the FF and the FFPE samples using the same variant caller. This is consistent with our findings on the metastatic prostate sample of patient UZ001. The difference between the variant callers in the FF sample was larger than the difference of each caller between the FF and the FFPE samples for GeL004, GeL300, GeL365 and UZ001 (see Suppl. Table 11**,** Additional File 1). In addition, for two tumor samples, the VAF was lower in the FFPE sample than in the FF sample (GeL007, GeL028), for one of them the opposite holds true (GeL008). For the rest of the tumor samples, the VAF was consistent between the FF and the FFPE samples (see Suppl. Table 12, Additional File 1). In general, Mutect2 and Strelka2 reported the most similar mutational profiles between the FF and the FFPE samples (see Suppl. Table 13, Additional File 1). No bias towards specific mutational patterns was observed in the FFPE samples.

**Table 9:** Summary table of the consistency (F1-scores) between the FF and the FFPE sample

Patient ID	Strelka2	Mutect2	VarScan2	Shimmer	Maximum	At least 2	Improvement
**UZ001**	0.6474	0.5167	0.3031	0.5349	0.6474	0.8290	0.1816
**GeL007**	0.1533	0.1563	0.0297	0.0061	0.1563	0.2317	0.0754
**GeL008**	0.2344	0.0823	0.0168	0.0023	0.2344	0.3895	0.1551
**GeL024**	0.2298	0.1756	0.0210	0.0107	0.2298	0.4203	0.1904
**GeL028**	0.3586	0.2485	0.0449	0.0335	0.3586	0.5326	0.1740
**GeL004**	0.5656	0.5226	0.1502	0.2200	0.5656	0.7149	0.1493
**GeL032**	0.0646	0.1012	0.0034	0.0004	0.1012	0.0647	−0.0365
**GeL065**	0.6308	0.5309	0.1604	0.0304	0.6308	0.6345	0.0037
**GeL300**	0.7724	0.7730	0.2358	0.5837	0.7730	0.8716	0.0986
**GeL365**	0.6532	0.6155	0.2473	0.1530	0.6532	0.8051	0.1519
**Average**	0.4310	0.3753	0.1212	0.1575	0.4350	0.5494	0.1144

#### Validation of the *at least two* strategy to call variants

To validate our *at least two* variant calling approach, we ran all four variant callers on the matched FF and FFPE samples for each patient. Then, we combined the variants using the *at least two* strategy that gave the best performance for the UZ001 patient. We calculated the F1-score between the variants called in the FF and the FFPE samples by each caller and compared them with the F1-score obtained when considering only variants reported by at least two variant callers in the FF and the FFPE sample. The results are shown in Table 9. For all samples, except GeL032, the variant calling approach significantly improves upon the best single caller score under default conditions. On average, more variants are called in the FFPE sample than in its FF counterpart, and this irrespective of the variant caller used (see Suppl. Table 14, Additional File 1). From that table it can also be seen that the one patient where the *at least two* strategy did not work, GeL032, has a disproportionate number of calls in the FFPE compared to the FF sample. While this is likely related to poor alignment quality in the FFPE sample, we decided for the sake of completeness to include the sample in all analyses, in line with the original publication of that sample [14].

Overall, the F1-score improves on average by 0.1144, suggesting that our variant calling strategy allows detecting robustly somatic SNVs in the FFPE samples, offering both a high sensitivity and precision. More detailed information about the sensitivity and precision of all variant callers on each sample are available in Suppl. Table 15 (Additional File 1). Note that selecting variants called by at least two callers also increases the correspondence of the mutational profiles between the FF and the FFPE samples (higher cosine similarity scores, see Suppl. Table 16, Additional File 1).

Analogously to the analysis we conducted for UZ001 patient, we also decomposed the mutational profiles of the 100,000 Genomes Project England samples into COSMIC signatures thereby assessing the contribution of sequencing artefact signatures (see **Methods**). As shown in Table 10, the contribution of artefact signatures to the FF and the FFPE mutational profiles was of the same order of magnitude for each of the paired FF-FFPE samples. All variant callers except VarScan2 reported a slightly higher proportion of artefact signatures for the FFPE sample. Interestingly, Table 10 shows that when considering only variants called by at least two variant callers, the proportion of signatures representative of artefacts (see Suppl. Table 1**,** Additional File 1) is significantly lower. This further confirms that our strategy successfully removes sequencing artefacts.

**Table 10:** Artefact contribution to the mutational profiles of the FF and the FFPE sample per caller. Values with an asterisk are artefact estimates based on reconstructed mutation profiles with a cosine similarity below 0.9 with the original mutation profiles. These may be unreliable (see **Methods**)

Patient ID	Strelka2		Mutect2		VarScan2		Shimmer		At least 2	
	*FF*	*FFPE*	*FF*	*FFPE*	*FF*	*FFPE*	*FF*	*FFPE*	*FF*	*FFPE*
**UZ001**	0.1419	0.0847	0.1024	0.0757	0.2010	0.0981	0.3252*	0.6759*	0.0822	0.0936
**GeL007**	0.1626	0.5381*	0.1285	0.1824	0.3843	0.1981	0.1555	0.1863	0.0714	0.1132
**GeL008**	0.2133	0.1377	0.1302	0.2501	0.4345	0.1925	0.2466*	0.1631	0.0796	0.1704
**GeL024**	0.4953	0.3111	0.1244	0.1261	0.5331	0.2191	0.1882	0.0556	0.1050	0.0899
**GeL028**	0.1344	0.1670	0.1041	0.0938	0.4947	0.1503	0.1152	0.0509	0.0666	0.0859
**GeL004**	0.2067	0.2896	0.1099	0.1611	0.3565	0.0932	0.1940	0.3090	0.0590	0.0775
**GeL032**	0.1164	0.1168	0.0833	0.2664	0.4955	0.1528	0.1474	0.3310	0.0747	0.0506
**GeL065**	0.1047	0.1686	0.0580	0.1127	0.1180	0.1425	0.1662	0.1458	0.1574	0.0816
**GeL300**	0.0822	0.2095	0.0781	0.0827	0.0583	0.0758	0.1022	0.1120	0.0544	0.0558
**GeL365**	0.0886	0.1701	0.0662	0.1192	0.0862	0.1051	0.0894	0.1362	0.0331	0.0436
**Mean**	**0.1746**	**0.2200**	**0.0985**	**0.1201**	**0.3162**	**0.1402**	**0.1730**	**0.2030**	**0.0783**	**0.0817**

### Assessing the extent to which intra-tumor heterogeneity (ITH) explains discrepancies in variants called between the FF and FFPE samples

Table 10 shows that the *at least two* variant calling strategy allows to reliably detect variants in most samples. By applying this strategy on both the FF and the FFPE samples, we can investigate why certain variants are unique to each sample type. Based on Fig. 1, the most plausible explanation would be ITH that results in sampling different subclonal variants. If ITH plays a key role in the difference between the FF and the FFPE samples, then we would expect that the subclonal variants show a lower overlap than the clonal ones. However, to properly define clonal and subclonal variants, it is necessary to correct the obtained VAFs for copy number (CN) status and sample purity.

To perform CN correction, the CN variant calling pipeline from GATK was used to identify regions that are amplified (+), deleted (−) or neutral (0) (see **Methods**). Although there is a limited number of publications demonstrating the feasibility of CN calling on FFPE, our results clearly showed a distinction between CN alterations called in the FF and the FFPE samples (see Suppl. Table 2**,** Additional File 1). The most plausible explanation for this observation is the presence of short CN altered regions that are only observed in the FFPE samples (see Suppl. Fig. 3**,** Additional File 2). It is highly improbable that these regions are effectively CN altered and therefore variants lying in these regions would be falsely corrected based on their CN status. To avoid introducing such additional bias, only variants called by at least two variants callers mapping to segments diploid in both the FF and the FFPE samples were kept (bona fide *variants*, see **Methods** and Suppl. Table 3**,** Additional File 1). Additionally, we also discarded variants located on the mitochondrial DNA and the sex chromosomes.

**Fig. 4:**
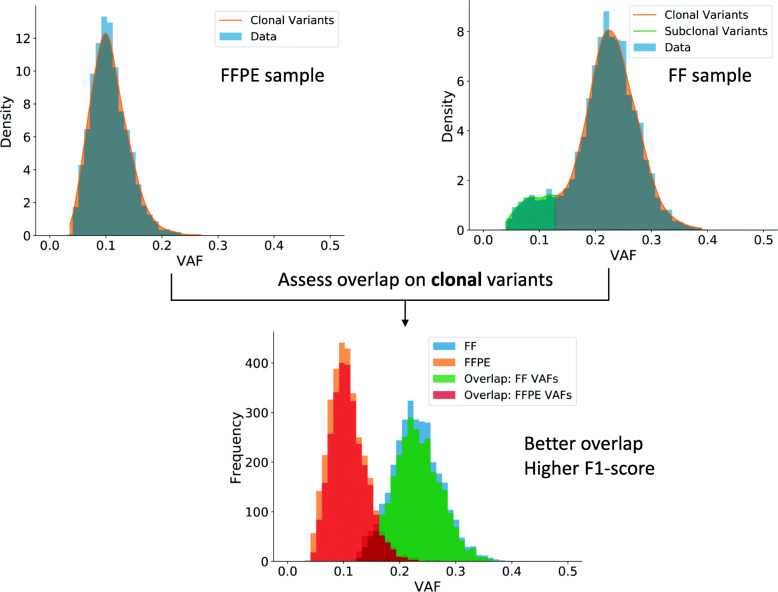
Evaluating the overlap between clonal variants from FF and FFPE samples of UZ001. By first defining the clonal and subclonal variants (see main text), it is possible to calculate the overlap between clonal variants only. Compared to Fig. 1, only variants using the at least two approach are shown, which are known to lie in diploid regions in both FF and FFPE and can be considered clonal. Overlap: red (green) refers to variants in the FFPE (FF) sample also found in the FF (FFPE) sample. Clearly, these additional filtering steps lead to an appreciable improvement in overlap between the FF and the FFPE sample

To estimate sample purity, we compared the pathologist purity estimates to those of two tools, TPES [21] and FACETS [22]. In general, the purity estimates for the same sample showed little consistency (see Suppl. Table 17, Additional File 1). Therefore, correcting for purity risks introducing yet another bias. Therefore, we aimed at identifying clonal variants for each sample individually, using a Kernel Density Estimation (KDE) approach (see **Methods**). For each sample, only considering bona fide diploid variants, the clonal variants are identified based on the VAF distribution. The KDE approach then allows finding the clonal population, regardless of the sample purity. Figure 4 illustrates the KDE approach on samples from UZ001 patient and demonstrates that the agreement between the FF and the FFPE sample is higher when considering only clonal variants.

Table 11 shows the overlap between clonal variants in the FF and the FFPE samples where variants were obtained using the *at least two* variant calling strategy. The results can be compared to subclonal variants (defined as all bona fide diploid variants that are not clonal). Note that, as expected, clonal variants are in general easier to retrieve than their subclonal counterparts. There is one example (GeL007) were the opposite holds, but further investigation showed that many clonal variants from the FF sample appeared as subclonal variants in the FFPE sample (see Suppl. Fig. 19, Additional File 2). These results demonstrate that some of the discrepancies between the FF and the FFPE samples from Table 10 can be attributed to ITH. Note that for some patients (e.g. patient UZ001, see Fig. 4), we completely lose the clonal structure in the FFPE sample, such that no subclonal variants can be defined in this sample. For each patient, we also explicitly verified how many subclonal variants in the FFPE sample were classified as clonal in the FF sample and vice versa (see Suppl. Table 18, Additional File 1). For most patients, the clonal structure in the FFPE sample agrees well with the FF sample, the most notable exception being samples from GeL032 patient.

**Table 11:** Number of clonal and subclonal variants detected in diploid regions common to FF and FFPE. For both clonal and subclonal variants the F1-score between the FF and the FFPE variants was calculated. Variants were called using the at least two variant calling strategy

Clonality	Clonal				Subclonal				At least 2
Patient ID	*FF*	*FFPE*	*Overlap*	*F1-score*	*FF*	*FFPE*	*Overlap*	*F1-score*	*F1-score*
**UZ001**	3229	3246	2795	0.86	372	0	0	0.00	0.83
**GeL007**	662	25	1	0.04	1862	1183	223	0.19	0.23
**GeL08**	1109	2417	996	0.41	425	0	0	0.00	0.39
**GeL024**	1016	296	244	0.82	275	1413	4	0.00	0.42
**GeL028**	1037	1585	908	0.57	721	0	0	0.00	0.53
**GeL004**	1074	1074	965	0.90	710	630	297	0.47	0.71
**GeL032**	3249	202	126	0.62	506	2103	4	0.00	0.06
**GeL065**	1434	1362	1287	0.94	587	552	321	0.58	0.63
**GeL300**	9660	9821	8890	0.91	354	678	16	0.02	0.87
**GeL365**	1099	1013	962	0.95	1331	1309	937	0.72	0.80

## Discussion

In this work, we have investigated whether a metastatic FFPE sample, embedded with recent protocols and subjected to DNA extraction using specialized procedures, can be used as a proxy for an FF sample to call somatic variants for cohort analysis. In contrast to previous studies, which focused on comparing the extent to which a small fraction of the most reliable variants compares between an FF and an FFPE sample (the fraction enriched in drivers or actionable mutations) [14], cohort analysis requires that as many true somatic variants as possible are called (high sensitivity) so that subsequent statistical analysis over a cohort can identify driver variants. Because of the subsequent statistical analysis, cohort analysis can tolerate some false positives and thus allows for a less stringent precision. Using four different variant callers (Strelka2, Mutect2, VarScan2 and Shimmer), we compared the somatic calls on the FFPE sample to its FF counterpart. At first sight, each variant caller recovered about 50% of the FF calls in the FFPE sample. Interestingly, we observed a larger discrepancy between variant callers on the same sample than between the FF and the FFPE sample using the same variant caller. This implies that the choice of variant calling tool is at least as important as whether FF or FFPE material is being used.

Using coverage versus VAF plots on the FF sample, a clear subpopulation of calls at VAF 0.25 was distinguishable and was enriched in highly significant calls, these were the calls we aimed to recover in the FFPE sample. However, while many of the calls detected in the FF were effectively present in the FFPE sample, they were reported with a lower VAF in the FFPE sample. This effect reduces the resolution of variant callers for the identification of low-VAF variants in the FFPE sample, reducing the overlap between the FF and FFPE samples. The effect was especially prominent in variant callers that rely on hypergeometric testing, i.e. VarScan2 and Shimmer. By choosing, for each variant caller, a specific threshold on the significance level of the identified variants, the overlap between the FF and the FFPE sample can be optimized in terms of sensitivity and precision. However, in many real-life situations, there is no matching FF sample available, and there is a need for a good strategy to perform a precise yet sensitive variant calling. Simply taking the intersection between the different callers, turned out to be too simplistic, as the low resolution of certain variant callers in the FFPE sample (in this case VarScan2 and Shimmer) obfuscated the final intersection. Indeed, for these two variants callers, the calls common to the FFPE sample and the FF gold standard are not receiving a more significant score. Nevertheless, when considering only calls reported by at least two variant callers (in our hands Strelka2, Mutect2, VarScan2 and Shimmer), we obtain almost 3700 calls present in both the FF and the FFPE samples, with an F1-score higher than 80%. Using the correct variant calling strategy, the overlap between the FF and FFPE sample in somatic SNVs increases to such an extent that a large fraction of the calls detected in the FFPE sample are contained in the FF sample and the number of variants unique to each sample remains restricted. In addition, the cosine similarity between the mutational profiles increased after selecting variants called by at least two callers. Moreover, this *at least two* variant callers strategy considerably reduces spurious calls related to sequencing artefacts.

The validation of our approach on nine paired FF-FFPE samples from the same tumors obtained from a pilot study of the 100,000 Genomes Project England demonstrated that the proposed variant calling strategy allows to robustly detect somatic SNVs in the FFPE samples. Indeed, our variant calling approach significantly improves upon the best single caller score, resulting in an F1-score that is on average 0.1144 higher. Finally, we demonstrated that discrepancies in variants detected in matching FF and FFPE samples can largely be attributed to ITH. Indeed, when focusing on clonal variants only, the average F1-score increased by another 0.1550, highlighting the very good overlap of clonal variants between the FF and the FFPE samples.

## Conclusion

In this study we aimed at investigating the feasibility of somatic SNV calling in FFPE material. While previous studies have focused on retrieving a set of highly reliable variants in FFPE using a matched FF sample, this study intended to maximize the overlap between the identified variants in matching FF and FFPE samples in the context of cohort analysis. Hereto, we developed and validated an optimized strategy for variant calling in FFPE. We could show that the strategy resulted in both a high sensitivity and precision but also in a better concordance between the mutational signatures of variants called in matching FF and FFPE. In general, the retrieved variants are clonal variants, implying that intra-tumor heterogeneity may make up a large fraction of the observed discrepancy between FF and FFPE samples. These observations all demonstrate that clonal SNVs can be accurately called using FFPE derived data, the most commonly available source for DNA material. In conclusion, studies that envisage driver identification based on cohort analysis can rely on FFPE material.

## Supplementary information

**Additional file 1: Supplementary Tables.** This document contains all the tables supplementary to the main manuscript.

**Additional file 2: Supplementary Figures.** This document contains all the figures supplementary to the main manuscript.

## Data Availability

The datasets generated during the current study are available in the European Genome-phenome Archive (EGA) repository, EGAS00001004456 (https://ega-archive.org/studies/EGAS00001004456). The data that support the findings of this study are available from the 100,000 Genomes Project England (GeL004, GeL007, GeL008, GeL024, GeL028, GeL032, GeL065, GeL300 and GeL365), but restrictions apply to the availability of these data, which were used under license for the current study. Data are however available from the authors upon reasonable request and with permission of 100,000 Genomes Project England.

## Abbreviations

AD: Allelic Depth
CK7: Cytokeratin 7
CN: Copy Number
CNV: Copy Number Variation
DNA: Deoxyribonucleic Acid
DP: Depth
CCF: Cancer Cell Fraction
FF: Fresh Frozen
FFPE: Formaldehyde Fixed-Paraffin Embedded
FWO: Fonds Wetenschappelijk Onderzoek-Vlaanderen
gDNA: Genomic DNA
GHU: Ghent University Hospital
H&E: Haematoxylin and Eosin
ITH: Intra-tumor Heterogeneity
IWT: Innovatie door Wetenschap en Technologie
KDE: Kernel Density Estimation
PoNs: Panel of Normals
PSA: Prostate Specific Antigen
NGS: Next Generation Sequencing
SBS: Single Base Substitution
SNV: Single Nucleotide Variation
TTF-1: Thyroid Transcription Factor 1
VAF: Variant Allele Frequency
WES: Whole Exome Sequencing
WGS: Whole Genome Sequencing
