# Supplementary material for: Comparative analysis of somatic variant calling on matched FF and FFPE WGS samples
Source: BMC Med Genomics. 2020 Jul 6;13:94. doi: 10.1186/s12920-020-00746-5 (PMC7336445; doi:10.1186/s12920-020-00746-5)
Supplement: Supplementary file 1 — Additional file 1: Supplementary Tables. This document contains all the tables supplementary to the main manuscript. [file 12920_2020_746_MOESM1_ESM.docx]

**Supplementary Tables**

**Supplementary Table 1.** COSMIC single nucleotide signatures (SBS) reported as possible sequencing artefacts.

| SBS27 | SBS43 | **SBS45** | SBS46 | SBS47 | SBS48 | SBS49 | SBS50 | SBS51 |
| --- | --- | --- | --- | --- | --- | --- | --- | --- |
| SBS52 | SBS53 | SBS54 | SBS55 | SBS56 | SBS57 | **SBS58** | SBS59 | SBS60 |

**Supplementary Table 2.** The percentage base pairs with a given copy number status, comparing FF with FFPE. For each status, the F1-score between the FF and the FFPE samples is given.

| **Patient ID** | **Percentage in FF** | | | **Percentage in FFPE** | | | **F1-score** | | |
| --- | --- | --- | --- | --- | --- | --- | --- | --- | --- |
|  | - | 0 | + | - | 0 | + | - | 0 | + |
| **UZ001** | 23.65 | 71.37 | 4.98 | 15.26 | 80.15 | 4.59 | 0.76 | 0.92 | 0.73 |
| **GeL007** | 1.11 | 98.69 | 0.20 | 0.43 | 93.59 | 5.99 | 0.22 | 0.95 | 0.06 |
| **GeL008** | 4.28 | 95.53 | 0.19 | 5.19 | 74.36 | 20.45 | 0.91 | 0.82 | 0.02 |
| **GeL024** | 0.26 | 99.64 | 0.10 | 3.69 | 75.33 | 20.98 | 0.10 | 0.77 | 0.01 |
| **GeL028** | 6.80 | 84.53 | 8.67 | 13.57 | 61.68 | 24.75 | 0.64 | 0.74 | 0.44 |
| **GeL004** | 34.74 | 64.48 | 0.78 | 34.90 | 57.24 | 7.86 | 0.96 | 0.90 | 0.10 |
| **GeL032** | 12.53 | 82.07 | 5.40 | 17.59 | 46.19 | 36.22 | 0.67 | 0.62 | 0.14 |
| **GeL065** | 35.06 | 52.07 | 12.87 | 26.67 | 41.69 | 31.64 | 0.71 | 0.73 | 0.60 |
| **GeL300** | 6.54 | 91.61 | 1.85 | 7.88 | 90.31 | 1.81 | 0.90 | 0.99 | 0.98 |
| **GeL365** | 24.48 | 50.35 | 25.17 | 41.69 | 33.62 | 24.69 | 0.70 | 0.77 | 0.73 |

**Supplementary Table 3.** Number of variants called by two callers, with a breakdown per copy number status. The last column denotes the number of variants that are considered bona fide diploid, I.e. variants that have a neutral copy number status in both FF and FFPE.

| **Patient ID** | **Type** | **Total** | **-** | **0** | **+** | **Bona fide** |
| --- | --- | --- | --- | --- | --- | --- |
| **UZ001** | FF | 4661 | 606 | 3704 | 346 | 3643 |
|  | FFPE | 4234 | 292 | 3672 | 269 | 3306 |
| **GeL007** | FF | 2873 | 30 | 2827 | 16 | 2526 |
|  | FFPE | 2073 | 19 | 1915 | 138 | 1263 |
| **GeL008** | FF | 2191 | 101 | 2085 | 5 | 1534 |
|  | FFPE | 6091 | 192 | 4322 | 1576 | 2418 |
| **GeL024** | FF | 1789 | 12 | 1773 | 4 | 1294 |
|  | FFPE | 3303 | 166 | 2526 | 605 | 1868 |
| **GeL028** | FF | 3285 | 160 | 2785 | 340 | 1762 |
|  | FFPE | 3313 | 377 | 2052 | 883 | 1591 |
| **GeL004** | FF | 3111 | 800 | 2277 | 34 | 1788 |
|  | FFPE | 3276 | 798 | 2164 | 306 | 1709 |
| **GeL032** | FF | 7852 | 531 | 6693 | 628 | 3763 |
|  | FFPE | 84340 | 3083 | 31005 | 50106 | 2548 |
| **GeL065** | FF | 7367 | 2651 | 3599 | 1116 | 2143 |
|  | FFPE | 11610 | 3434 | 5875 | 2291 | 2020 |
| **GeL300** | FF | 11678 | 403 | 10997 | 278 | 10279 |
|  | FFPE | 12304 | 536 | 11496 | 270 | 10764 |
| **GeL365** | FF | 7443 | 1491 | 3973 | 1979 | 2431 |
|  | FFPE | 7564 | 2531 | 2675 | 2355 | 2323 |

**Supplementary Table 4.** Mutation metrics of C>T substitutions in FFPE and FF samples (UZ001).

|  | **FFPE** | **FF** |
| --- | --- | --- |
| C>T mutation % | 25.02 | 24.92 |
| C>T mutation count | 4883 | 4885 |
| Total amount of mutations | 19516 | 19603 |

**Supplementary Table 5.** Quality control (QC) metrics for FFPE, FF and normal (blood) samples (UZ001).

| **QC metric** | **FFPE** | **FF** | **Blood** |
| --- | --- | --- | --- |
| % GC content (average of R1 and R2) | 39 | 40 | 42 |
| % Duplicate Reads | 20.62 | 20.08 | 16.78 |
| Mean Quality Score (Phred Score) | 38.86 | 37.73 | 39.25 |
| % Adaptor Content | 0 | 0 | 0.01 |
| Bases (Gigabases) | 449.05 | 436.53 | 128.95 |
| Reads (Million) | 2988.51 | 2902.05 | 857.37 |
| % Bases in R1 with Phred quality score >= 30 (% Bases with Q30) | 95.94 | 95.74 | 96.57 |
| % Bases in R2 with Phred quality score >=30 (% Bases with Q30) | 92 | 86.65 | 94.05 |
| % mapped reads | 99.59 | 95.92 | 99.85 |
| Mean Coverage | 107.38 | 106.08 | 28.98 |
| Minimum Coverage: % of Bases at 10X | 99.55 | 99.57 | 95.62 |
| Minimum Coverage: % of Bases at 20X | 99.35 | 99.39 | 77.36 |
| Minimum Coverage: % of Bases at 30X | 99.11 | 99.15 | 43.96 |
| % reads trimmed | 0.75 | 0.22 | 0.19 |
| % reads remained after trimming adaptor | 99.99 | 99.99 | 99.99 |
| Total amount (µg) | 1.80560 | 0.64960 | 3.69900 |

**Supplementary Table 6.** Mutual overlap of variant callers for samples of patient UZ001. Diagonal = overlap between the FF and the FFPE sample for each caller. Upper triangle (orange) = overlap between callers for the FF sample. Lower triangle (green) = overlap between callers for the FFPE sample.

|  | **Strelka2** | **Mutect2** | **VarScan2** | **Shimmer** |
| --- | --- | --- | --- | --- |
| **Strelka2** | in FF: 6292  in FFPE: 6761  common: **4225** | 4410 | 2856 | 623 |
| **Mutect2** | 4065 | in FF: 10460  in FFPE: 11815  common: **5755** | 2990 | 766 |
| **VarScan2** | 358 | 464 | in FF: 4067  in FFPE: 1760  common: **883** | 956 |
| **Shimmer** | 16 | 69 | 79 | in FF: 8109  in FFPE: 10080  common: **4865** |

**Supplementary Table 7.** Proportion of calls reported by other callers for each variant caller in samples (UZ001).

| **FF sample** | **Mutect2** | **Strelka2** | **VarScan2** | **Shimmer** |
| --- | --- | --- | --- | --- |
| Not reported by any other callers | 6003 | 1596 | 992 | 7302 |
| Total calls | 10460 | 6292 | 4067 | 8109 |
| *% calls reported by other callers* | *42.61%* | *74.99%* | *75.61%* | *9.95%* |
| **FFPE sample** | **Mutect2** | **Strelka2** | **VarScan2** | **Shimmer** |
| Not reported by any other callers | 7720 | 2529 | 1226 | 9958 |
| Total calls | 11815 | 6761 | 1760 | 10080 |
| *% calls reported by other callers* | *34.66%* | *62.59%* | *30.34%* | *1.21%* |

***Supplementary Table 8.*** *Cosine similarity between mutational profiles of the FF and the FFPE sample (UZ001).*

|  | **Strelka2** | **Mutect2** | **VarScan2** | **Shimmer** | **Average** | **Std. Dev.** |
| --- | --- | --- | --- | --- | --- | --- |
| **UZ001** | 0.977 | 0.991 | 0.914 | 0.646 | 0.8820 | 0.1609 |

**Supplementary table 9.** Proportion of variants present in dbSNP database (=common germline variants) for each caller (UZ001).

| **Caller** | **Sample** | **In dbSNP** | **VAF < 0.5** | **%** | **In dbSNP** | **VAF > 0.5** | **%** | **Ratio of %** |
| --- | --- | --- | --- | --- | --- | --- | --- | --- |
| **Strelka2** | FF | 604 | 6082 | 9.93 | 163 | 210 | 77.62 | 7.82 |
|  | FFPE | 899 | 6719 | 13.38 | 32 | 42 | 76.19 | 5.69 |
| **Mutect2** | FF | 592 | 10412 | 5.69 | 3 | 48 | 6.25 | 1.10 |
|  | FFPE | 683 | 11808 | 5.78 | 2 | 7 | 28.57 | 4.94 |
| **VarScan2** | FF | 305 | 3637 | 8.39 | 282 | 430 | 65.81 | 7.84 |
|  | FFPE | 225 | 1379 | 16.32 | 263 | 381 | 69.03 | 4.23 |
| **Shimmer** | FF | 305 | 7346 | 4.15 | 305 | 763 | 39.97 | 9.65 |
|  | FFPE | 204 | 9329 | 2.19 | 172 | 751 | 22.90 | 10.45 |

**Supplementary Table 10.** Spearman correlations between significance scores of common variants (UZ001). Diagonal = correlations of variants reported in both the FF and the FFPE sample for each caller. Upper triangle (orange) = correlations of variants reported in the FF sample by pair of callers. Lower triangle (green) = correlations of variants reported in the FFPE sample by pair of callers. A significance level of 0.005 was imposed after Bonferroni correction for multiple hypothesis testing (p-value of 0.0003) (in red).

|  | **Strelka2** | **Mutect2** | **VarScan2** | **Shimmer** |
| --- | --- | --- | --- | --- |
| **Strelka2** | **0.5096**  4.37e-278 | **0.4430**  2.12e-211 | **0.1340**  1.90e-13 | **-0.0314**  4.29e-1 |
| **Mutect2** | **0.4791**  2.10e-232 | **0.4398**  6.12e-271 | **-0.3611**  1.09e-88 | **-0.3169**  8.20e-16 |
| **VarScan2** | **0.0859**  6.45e-2 | **-0.3812**  7.97e-14 | **0.1145**  6.54e-4 | **0.7945**  4.12e-140 |
| **Shimmer** | **-0.2784**  2.63e-1 | **-0.6000**  8.76e-1 | **0.6413**  9.74e-4 | **0.2310**  5.61e-2 |

**Supplementary Table 11.** Average and maximal F1-scores of three comparisons. (1) When comparing somatic variants called on different samples with the same variant caller. (2) When comparing somatic variants called by different variant callers on the FF sample. (3) When comparing somatic variants called by different variant callers on the FFPE sample.

| **Patient ID** | **Average F1-score between samples for the same caller (1)** | **Max F1-score between samples for the same caller (1)** | **Average F1-score between callers for the FF sample (2)** | **Max F1-score between callers for the FF sample (2)** | **Average F1-score between callers for the FFPE sample (3)** | **Max F1-score between callers for the FFPE sample (3)** |
| --- | --- | --- | --- | --- | --- | --- |
| **UZ001** | 0.3988 | 0.6474 | 0.3411 | 0.5773 | 0.1047 | 0.4375 |
| **GeL007** | 0.0863 | 0.1563 | 0.1337 | 0.6092 | 0.0406 | 0.1600 |
| **GeL008** | 0.0839 | 0.2344 | 0.1459 | 0.5792 | 0.0432 | 0.1265 |
| **GeL024** | 0.1093 | 0.2298 | 0.1974 | 0.5315 | 0.0588 | 0.2365 |
| **GeL028** | 0.1714 | 0.3586 | 0.3351 | 0.6447 | 0.0706 | 0.2930 |
| **GeL004** | 0.3646 | 0.5656 | 0.2775 | 0.5475 | 0.1592 | 0.4338 |
| **GeL032** | 0.0424 | 0.1012 | 0.4556 | 0.8532 | 0.0597 | 0.1899 |
| **GeL065** | 0.3381 | 0.6308 | 0.3881 | 0.8321 | 0.1256 | 0.4570 |
| **GeL300** | 0.5912 | 0.7730 | 0.3920 | 0.8558 | 0.4422 | 0.7490 |
| **GeL365** | 0.4173 | 0.6532 | 0.3926 | 0.7678 | 0.1652 | 0.5588 |

**Supplementary Table 12.** VAF median of calls common to the FF and the FFPE samples. Only variants called by at least two callers were considered.

| Patient ID | FF | FFPE | Difference |
| --- | --- | --- | --- |
| UZ001 | 0.2323 | 0.1083 | 0.1240 |
| GeL007 | 0.2039 | 0.0961 | 0.1078 |
| GeL008 | 0.2162 | 0.3194 | -0.1032 |
| GeL024 | 0.2542 | 0.2791 | -0.0249 |
| GeL028 | 0.3662 | 0.2432 | 0.1230 |
| GeL004 | 0.4158 | 0.3708 | 0.0450 |
| GeL032 | 0.2703 | 0.2500 | 0.0203 |
| GeL065 | 0.3947 | 0.3333 | 0.0614 |
| GeL300 | 0.2680 | 0.3158 | -0.0478 |
| GeL365 | 0.3818 | 0.3684 | 0.0134 |

***Supplementary Table 13.*** *Cosine similarity between mutational signature profiles in the FF and the FFPE samples.*

| **Patient ID** | **Strelka2** | **Mutect2** | **VarScan2** | **Shimmer** | **Average** | **Std. Dev.** |
| --- | --- | --- | --- | --- | --- | --- |
| **UZ001** | 0.977 | 0.991 | 0.914 | 0.646 | 0.8820 | 0.1609 |
| **GeL007** | 0.569 | 0.927 | 0.840 | 0.641 | 0.7442 | 0.1673 |
| **GeL008** | 0.881 | 0.818 | 0.795 | 0.590 | 0.7710 | 0.1260 |
| **GeL024** | 0.941 | 0.941 | 0.797 | 0.707 | 0.8465 | 0.1151 |
| **GeL028** | 0.917 | 0.942 | 0.816 | 0.784 | 0.8647 | 0.0766 |
| **GeL004** | 0.959 | 0.983 | 0.906 | 0.848 | 0.9240 | 0.0600 |
| **GeL032** | 0.980 | 0.987 | 0.774 | 0.721 | 0.8655 | 0.1380 |
| **GeL065** | 0.995 | 0.998 | 0.990 | 0.877 | 0.9650 | 0.0588 |
| **GeL300** | 0.968 | 0.995 | 0.936 | 0.986 | 0.9712 | 0.0127 |
| **GeL365** | 0.978 | 0.985 | 0.981 | 0.950 | 0.9735 | 0.0159 |
| **Mean** | 0.9165 | 0.9567 | 0.8749 | 0.7750 | 0.8808 | 0.0931 |

**Supplementary Table 14.** Ratio of somatic variants reported in FFPE and FF sample for each variant caller.

| **Patient ID** | **Strelka2** | **Mutect2** | **VarScan2** | **Shimmer** | **At least 2** |
| --- | --- | --- | --- | --- | --- |
| **UZ001** | 1.07 | 1.13 | 0.43 | 1.24 | 1.10 |
| **GeL004** | 1.12 | 1.29 | 4.69 | 1.54 | 1.05 |
| **GeL007** | 1.59 | 2.08 | 5.86 | 17.59 | 0.72 |
| **GeL008** | 4.20 | 11.09 | 25.36 | 32.25 | 2.78 |
| **GeL024** | 2.32 | 4.25 | 21.06 | 6.29 | 1.85 |
| **GeL028** | 1.58 | 2.36 | 2.70 | 3.39 | 1.01 |
| ***GeL032*** | *11.90* | *7.47* | *71.71* | *81.00* | *10.74* |
| **GeL065** | 1.51 | 2.06 | 3.28 | 1.74 | 1.58 |
| **GeL300** | 1.22 | 1.08 | 5.34 | 1.81 | 1.05 |
| **GeL365** | 1.36 | 1.32 | 2.31 | 0.67 | 1.02 |
| **Total** | 2.79 | 3.41 | 14.27 | 14.75 | 2.29 |
| **Total without GeL032** | 1.78 | 2.96 | 7.89 | 7.39 | **1.35** |

**Supplementary Table 15.** Performance measures of calls considering the FF sample as gold standard for each variant caller.

| **Patient ID** | | **GeL004** | **GeL007** | **GeL008** | **GeL024** | **GeL028** | **GeL032** | **GeL065** | **GeL300** | **GeL365** |
| --- | --- | --- | --- | --- | --- | --- | --- | --- | --- | --- |
| **Strelka2** | Overlap | *3446* | *719* | *1885* | *1232* | *1942* | *3654* | *6515* | *11173* | *6842* |
|  | Sens. | 0.6000 | 0.1984 | 0.6094 | 0.3817 | 0.4630 | 0.4165 | 0.7910 | 0.8589 | 0.7691 |
|  | Prec. | 0.5349 | 0.1249 | 0.1451 | 0.1644 | 0.2926 | 0.0350 | 0.5245 | 0.7017 | 0.5676 |
|  | F1 | 0.5656 | 0.1533 | 0.2344 | 0.2298 | 0.3586 | 0.0646 | 0.6308 | 0.7724 | 0.6532 |
| **Mutect2** | Overlap | *3080* | *1371* | *2153* | *1526* | *2302* | *3773* | *6458* | *10736* | *6695* |
|  | Sens. | 0.5976 | 0.2409 | 0.4979 | 0.4607 | 0.4176 | 0.4288 | 0.8123 | 0.8046 | 0.7126 |
|  | Prec. | 0.4643 | 0.1156 | 0.0449 | 0.1085 | 0.1769 | 0.0574 | 0.3943 | 0.7438 | 0.5418 |
|  | F1 | 0.5226 | 0.1563 | 0.0823 | 0.1756 | 0.2485 | 0.1012 | 0.5309 | 0.773 | 0.6155 |
| **VarScan2** | Overlap | *610* | *116* | *223* | *120* | *201* | *234* | *3197* | *854* | *1823* |
|  | Sens. | 0.4272 | 0.1019 | 0.2206 | 0.2317 | 0.0832 | 0.1219 | 0.3435 | 0.7472 | 0.4087 |
|  | Prec. | 0.0911 | 0.0174 | 0.0087 | 0.011 | 0.0308 | 0.0017 | 0.1046 | 0.14 | 0.1773 |
|  | F1 | 0.1502 | 0.0297 | 0.0168 | 0.021 | 0.0449 | 0.0034 | 0.1604 | 0.2358 | 0.247 |
| **Shimmer** | Overlap | *856* | *36* | *26* | *51* | *186* | *111* | *381* | *5714* | *758* |
|  | Sens. | 0.2792 | 0.0563 | 0.0387 | 0.039 | 0.0736 | 0.0162 | 0.0417 | 0.819 | 0.1277 |
|  | Prec. | 0.1815 | 0.0032 | 0.0012 | 0.0062 | 0.0217 | 0.0002 | 0.0239 | 0.4532 | 0.1907 |
|  | F1 | 0.2200 | 0.0061 | 0.0023 | 0.0107 | 0.0335 | 0.0004 | 0.0304 | 0.5837 | 0.153 |
| **At least 2** | Overlap | *2283* | *573* | *1613* | *1070* | *1757* | *2981* | *6020* | *10451* | *6041* |
|  | Sens. | 0.7338 | 0.1994 | 0.7362 | 0.5981 | 0.5349 | 0.379 | 0.8172 | 0.8949 | 0.8116 |
|  | Prec. | 0.6969 | 0.2764 | 0.2648 | 0.3239 | 0.5303 | 0.0353 | 0.5185 | 0.8494 | 0.7987 |
|  | F1 | 0.7149 | 0.2317 | 0.3895 | 0.4203 | 0.5326 | 0.0647 | 0.6345 | 0.8716 | 0.8051 |

***Supplementary Table 16.*** *Cosine similarity between mutational signature profiles of the FF and the FFPE samples. Only variants reported by at least two callers were considered.*

| **UZ001** | **GeL007** | **GeL008** | **GeL024** | **GeL028** | **GeL004** | **GeL032** | **GeL065** | **GeL300** | **GeL365** |
| --- | --- | --- | --- | --- | --- | --- | --- | --- | --- |
| 0.995 | 0.927 | 0.853 | 0.968 | 0.982 | 0.983 | 0.717 | 0.968 | 0.999 | 0.995 |

**Supplementary Table 17.** Tumor purities as estimated by different methods. <low> denotes a low purity sample. Note: EPIC could only be run on UZ001 as it had matching RNA data.

| **Patient ID** | **FF** | | | | **FFPE** | | | |
| --- | --- | --- | --- | --- | --- | --- | --- | --- |
|  | *Pathologist* | *FACETS* | *TPES* | *EPIC* | *Pathologist* | *FACETS* | *TPES* | *EPIC* |
| **UZ001** | >70 | 44 | 95 | 89 | >70 | 29 | 93 | 89 |
| **GeL007** | 60 | 30 | 45 | / | 60 | 29 | 21 | / |
| **GeL008** | 50 | 38 | 46 | / | 50 | 40 | 63 | / |
| **GeL024** | >40 | <low> | 54 | / | 60 | 18 | 29 | / |
| **GeL028** | >40 | 70 | 75 | / | 70 | 27 | 48 | / |
| **GeL004** | 100 | 85 | 90 | / | 95 | 80 | 82 | / |
| **GeL032** | 95 | 56 | 55 | / | 95 | 32 | 96 | / |
| **GeL065** | 95 | 42 | 93 | / | 95 | 62 | 95 | / |
| **GeL300** | >40 | 55 | 54 | / | >40 | 60 | 65 | / |
| **GeL365** | 95 | 65 | 94 | / | 95 | 68 | 95 | / |

**Supplementary Table 18.** Comparison of the clonal structure. For both the FF and FFPE sample, we identified clonal and subclonal variants (see main text) and compared the clonal status for each of the overlapping bona fide diploid variants. All these common variants received a label, either ‘clonal’ or ‘subclonal’ and the F1-score between the two was calculated. A high F1-score implies that clonal variants in FF are consistently labelled as clonal in FFPE.

|  | UZ001 | GeL007 | GeL008 | GeL024 | GeL028 | GeL004 | GeL032 | GeL065 | GeL300 | GeL265 |
| --- | --- | --- | --- | --- | --- | --- | --- | --- | --- | --- |
| F1-score | 0.97 | 0.45 | 0.87 | 0.38 | 0.98 | 0.95 | 0.11 | 0.96 | 1.00 | 0.95 |
