# Supplementary material for: Comparative analysis of somatic variant calling on matched FF and FFPE WGS samples
Source: BMC Med Genomics. 2020 Jul 6;13:94. doi: 10.1186/s12920-020-00746-5 (PMC7336445; doi:10.1186/s12920-020-00746-5)
Supplement: Supplementary file 2 — Additional file 2: Supplementary Figures. This document contains all the figures supplementary to the main manuscript. [file 12920_2020_746_MOESM2_ESM.docx]

**Supplementary Figures**


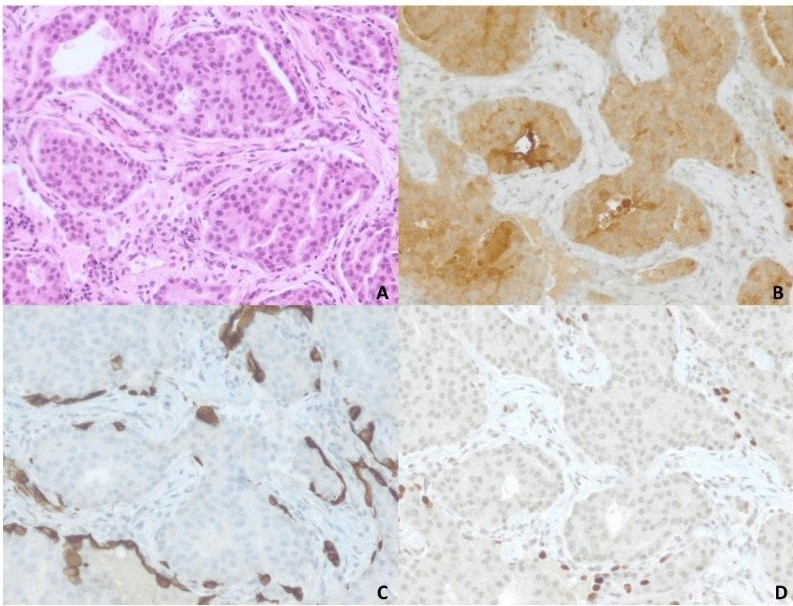


**Supplementary Figure 1.** Histopathology of the prostate cancer metastasis to the lung of patient UZ001. Panel A: stained with H&E, panel B: stained with prostate specific antigen (PSA), panel C: stained with thyroid transcription factor 1 (TTF-1) and panel D: stained with cytokeratin 7 (CK7). Note the strong positive staining for PSA in the tumor cells (B), while there is a lack of staining with CK7 (C) and TTF-1 (D). All images are 40X.

**
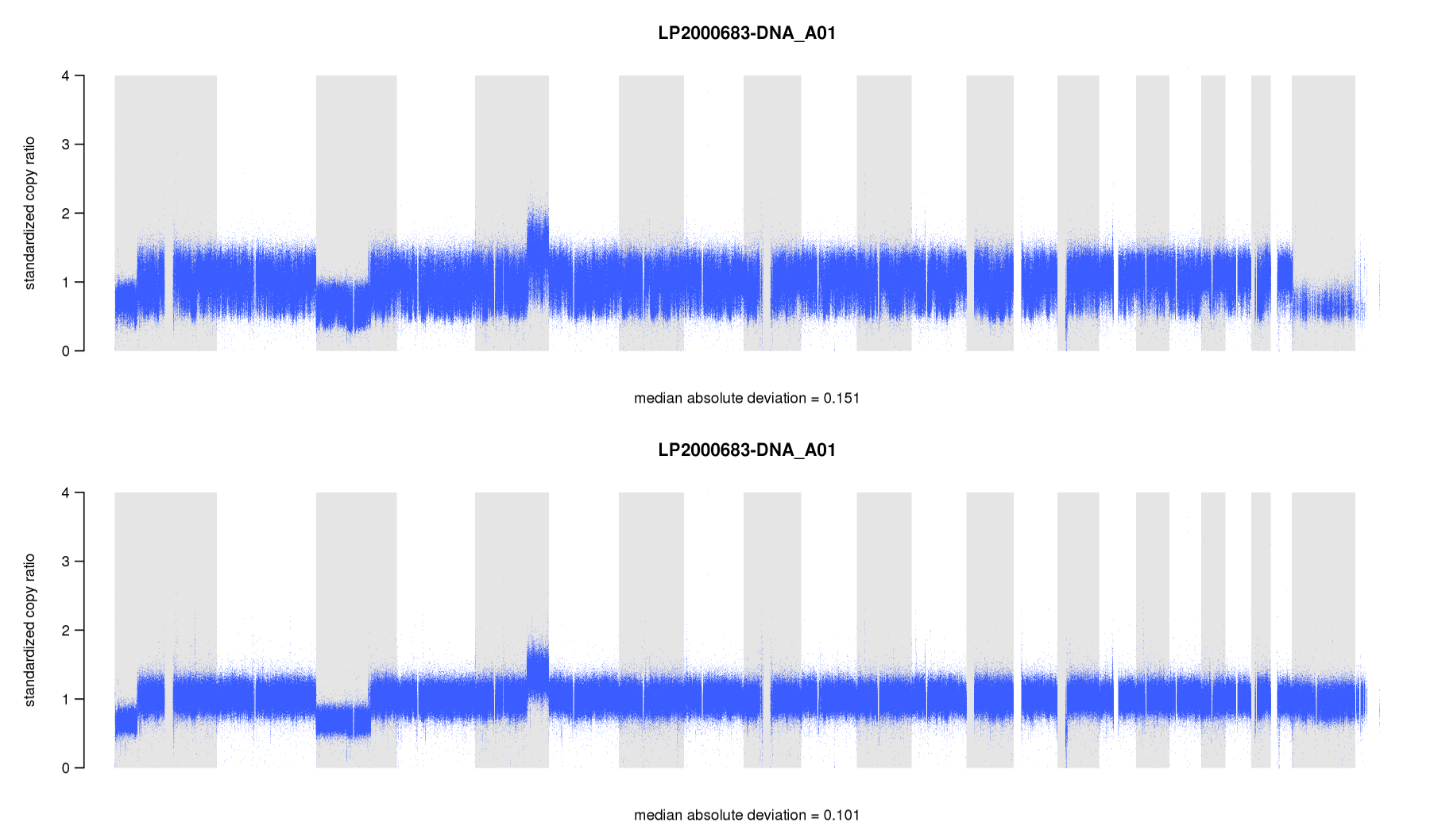
Supplementary Figure 2.** Denoised read counts for the FFPE sample of patient GeL365. Upper panel is before and the lower panel is after PoNs correction.


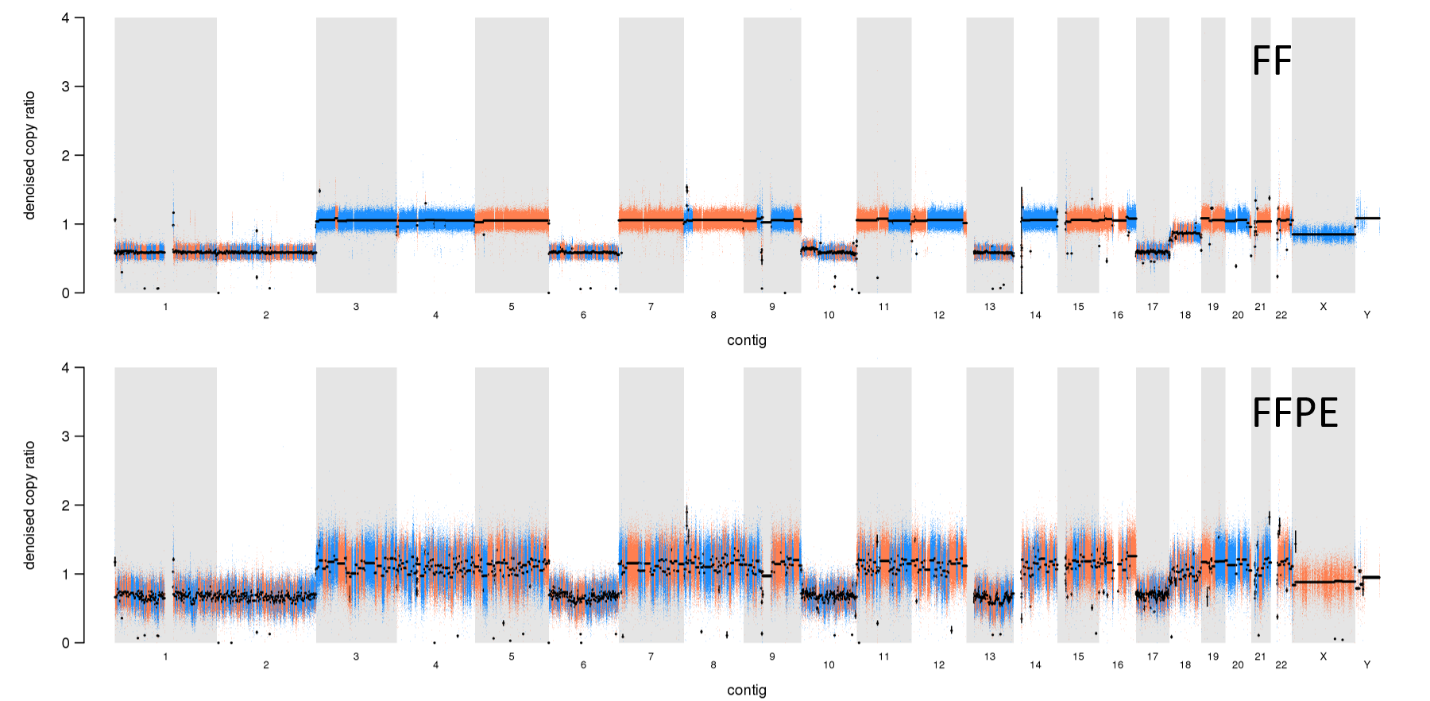


**Supplementary Figure 3.** CNV segments called on the FF and the FFPE samples from patient GeL004. On nearly every sample we could observe shattered segments in the FFPE samples, that are very likely to be non-biological. Because sometimes these shattered segments are falsely assigned a CN alteration, we only considered variants that are in diploid regions in both the FF and FFPE sample.


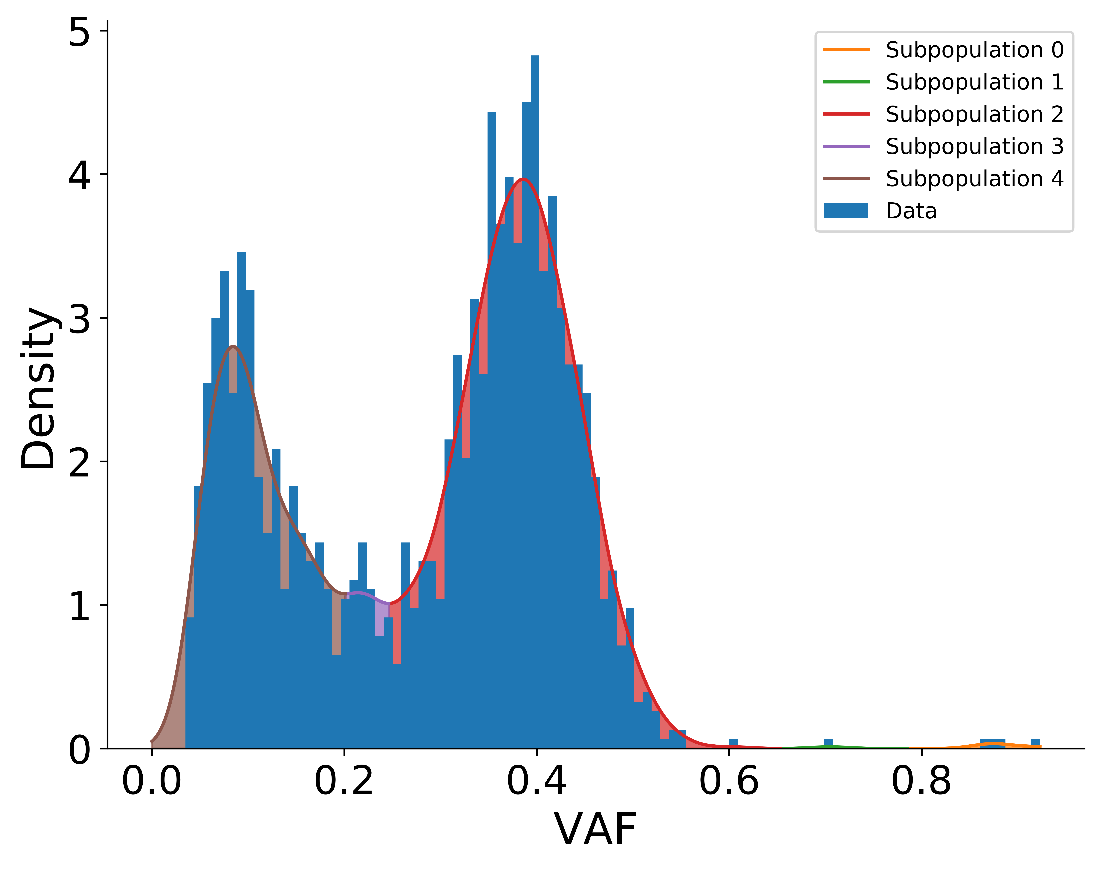


**Supplementary Figure 4.** KDE partitioning of the VAF distribution of the FFPE sample of patient GeL004. At every local minimum of the density, a cut is made, illustrated by a color transition. Each partition loosely corresponds to a different subclonal population, where in this example all variants belonging to the red peak (‘Subpopulation 2’) would be labelled clonal variants, based upon visual inspection.


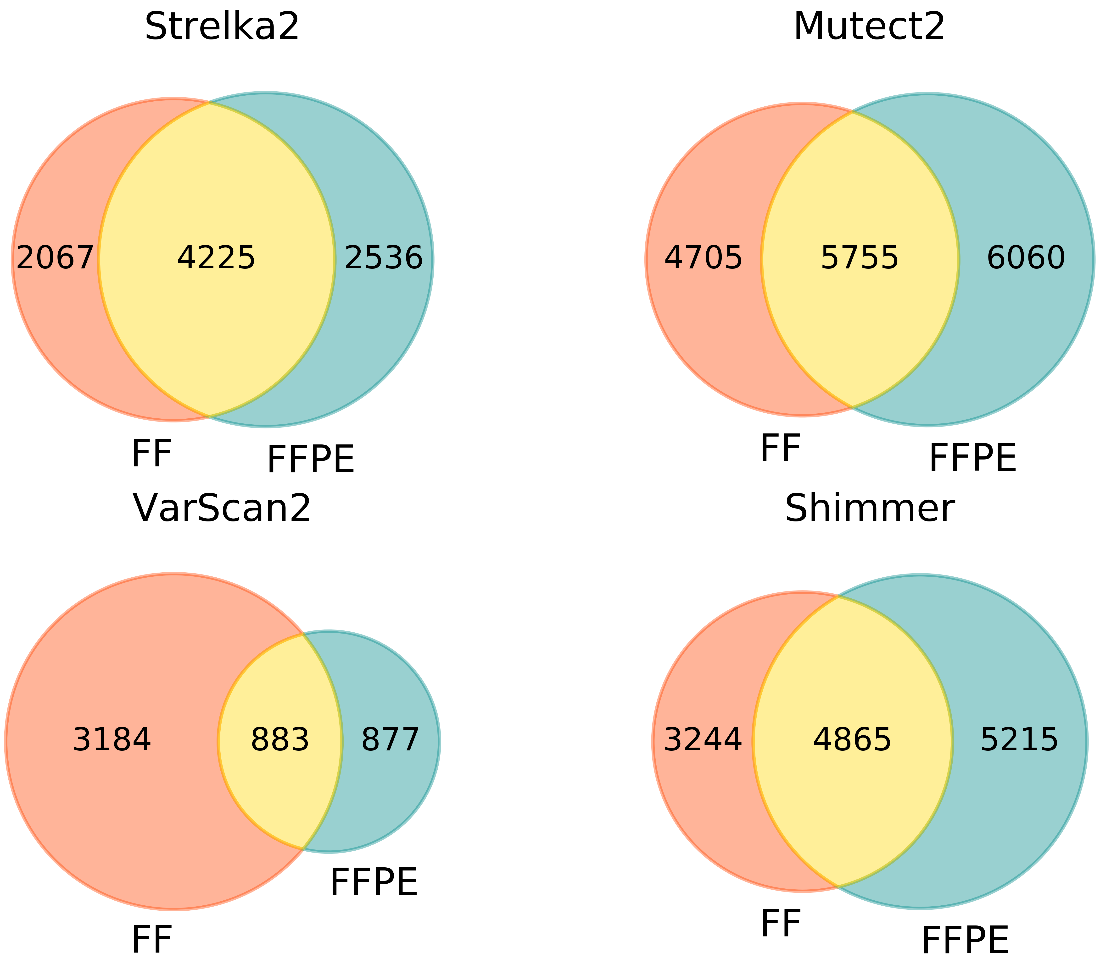


**Supplementary Figure 5.** Venn diagrams of somatic variants reported in the FF and the FFPE sample per caller (UZ001). Top left: using Strelka2, top right: using Mutect2 and applying FilterMutectCalls tool, bottom left: using VarScan2 and applying somaticFilter tool and bottom right: using Shimmer.


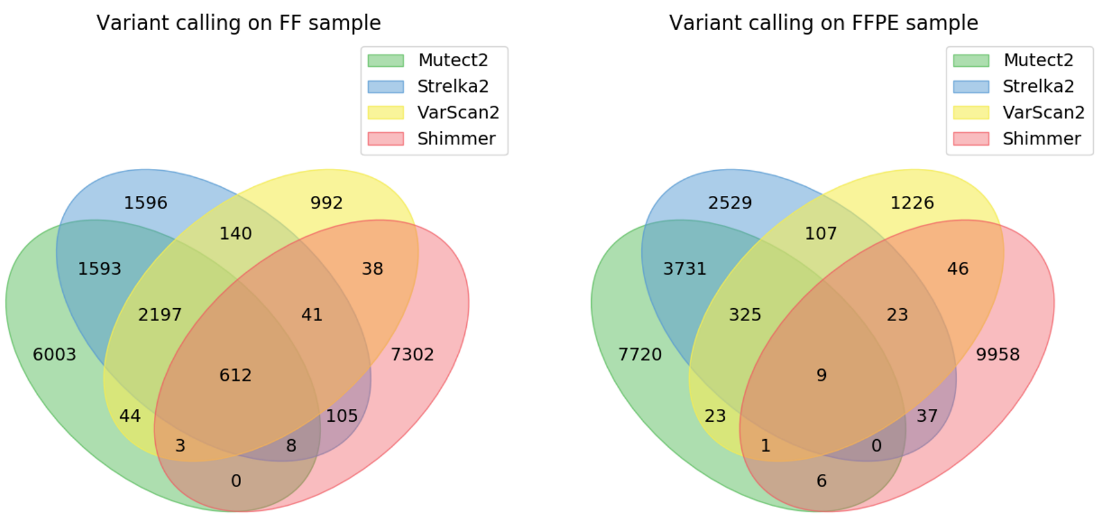


**Supplementary Figure 6.** Venn diagrams of somatic variants reported by every callers per sample (UZ001). FilterMutectCalls was applied on the Mutect2 output and somaticFilter on the VarScan2 output.


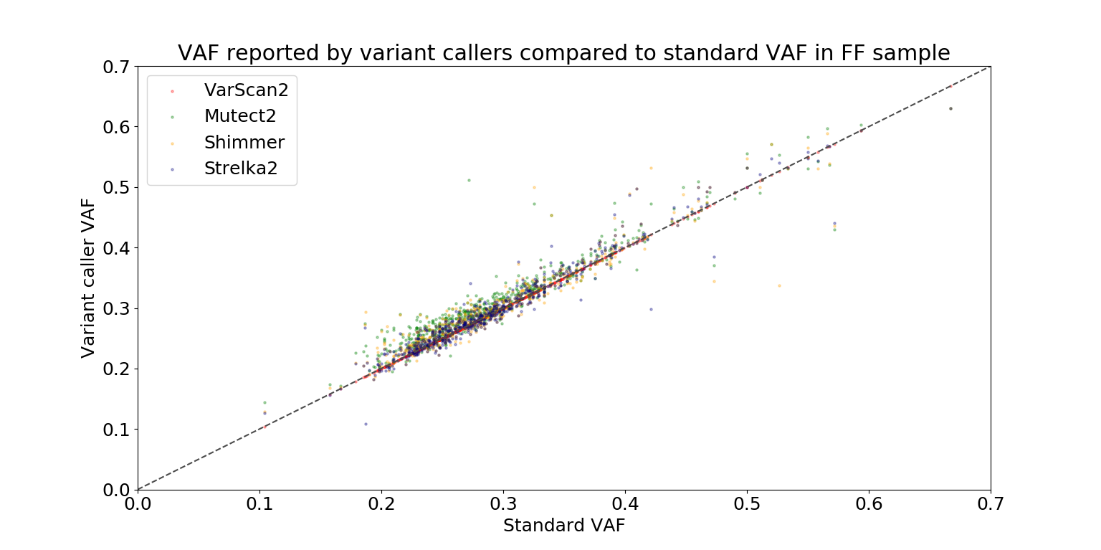


**Supplementary Figure 7.** Comparison of the VAF for variants returned by all the callers in FF sample (UZ001). Red = VarScan2, green = Mutect2, orange = Shimmer and blue = Strelka2.


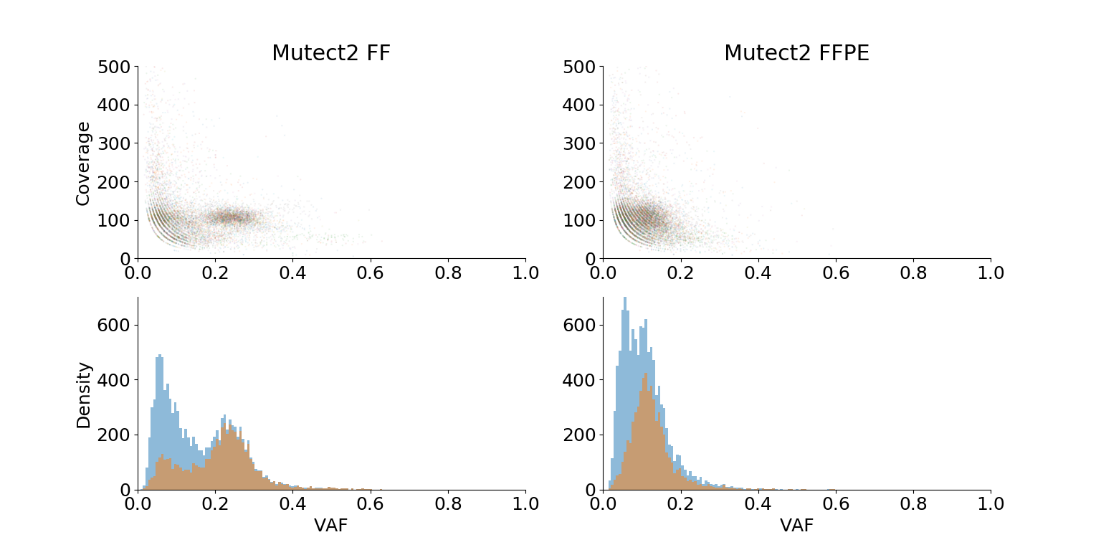


**Supplementary Figure 8.** (Sub)clonal population identification in variants reported in the FF and the FFPE sample by Mutect2 (UZ001). The subpopulations can be identified by distinct peaks in the distribution of the VAFs as obtained by Mutect2.


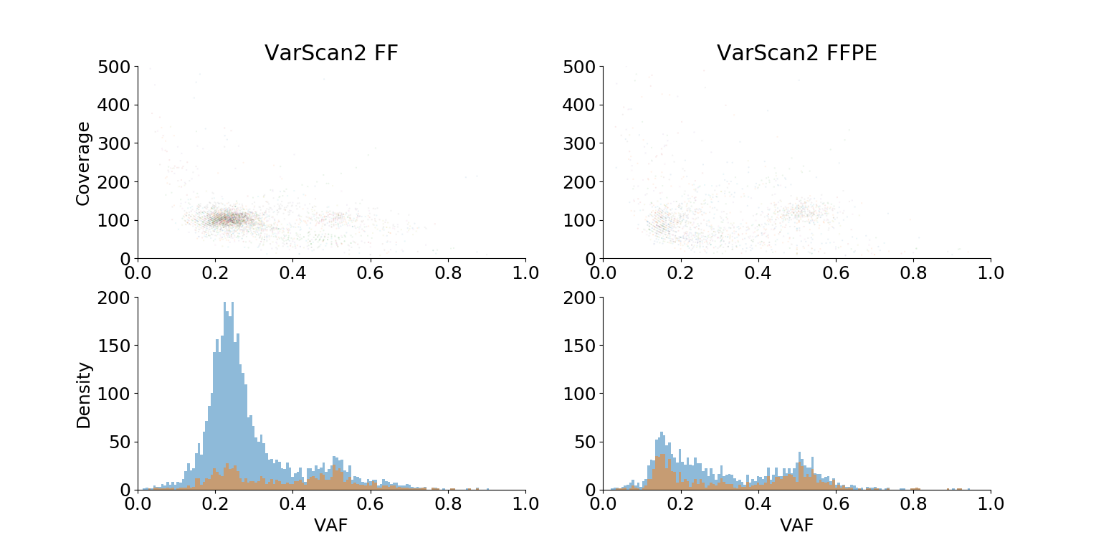

**Supplementary Figure 9.** (Sub)clonal population identification in variants reported in the FF and the FFPE sample by VarScan2 (UZ001). The subpopulations can be identified by distinct peaks in the distribution of the VAFs as obtained by VarScan2.


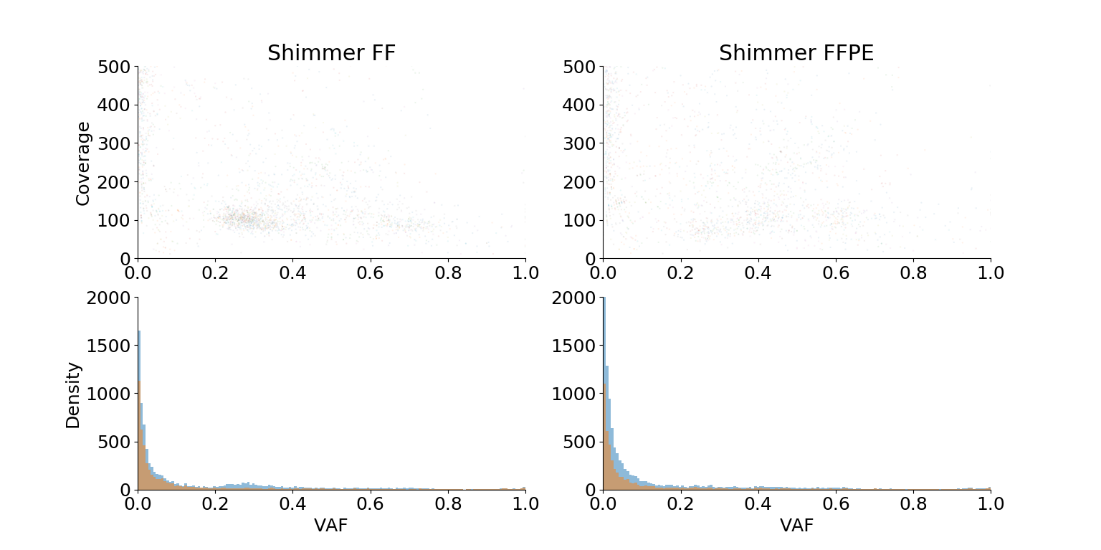


**Supplementary Figure 10.** (Sub)clonal population identification in variants reported in the FF and the FFPE sample by Shimmer (UZ001). The subpopulations can be identified by distinct peaks in the distribution of the VAFs as obtained by Shimmer.


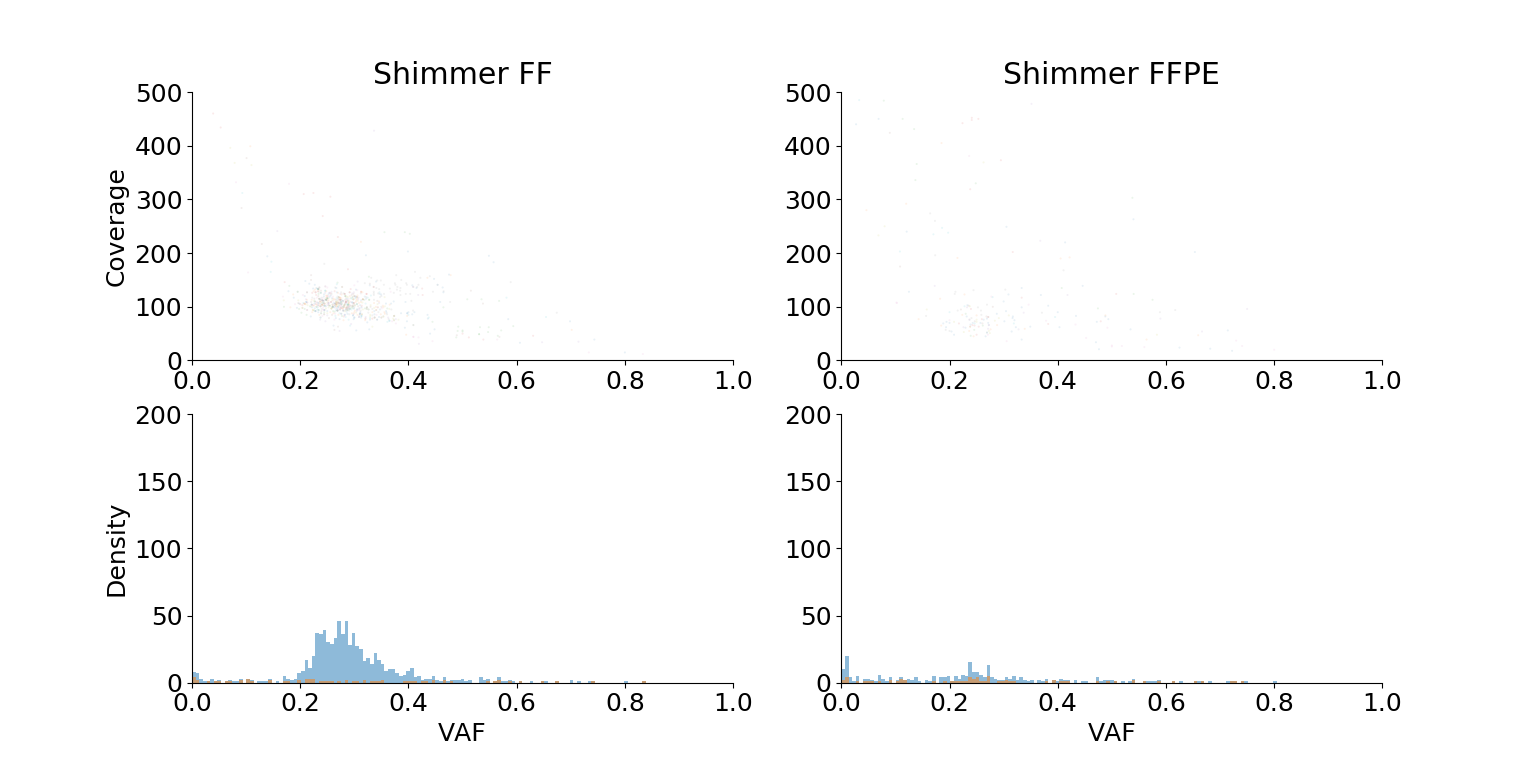


**Supplementary Figure 11.** (Sub)clonal population of variants reported in the FF and the FFPE sample by Shimmer (UZ001). The subpopulations can be identified by distinct peaks in the distribution of the VAFs as obtained by Shimmer with imposing a threshold of VAF of 0 in the normal sample.


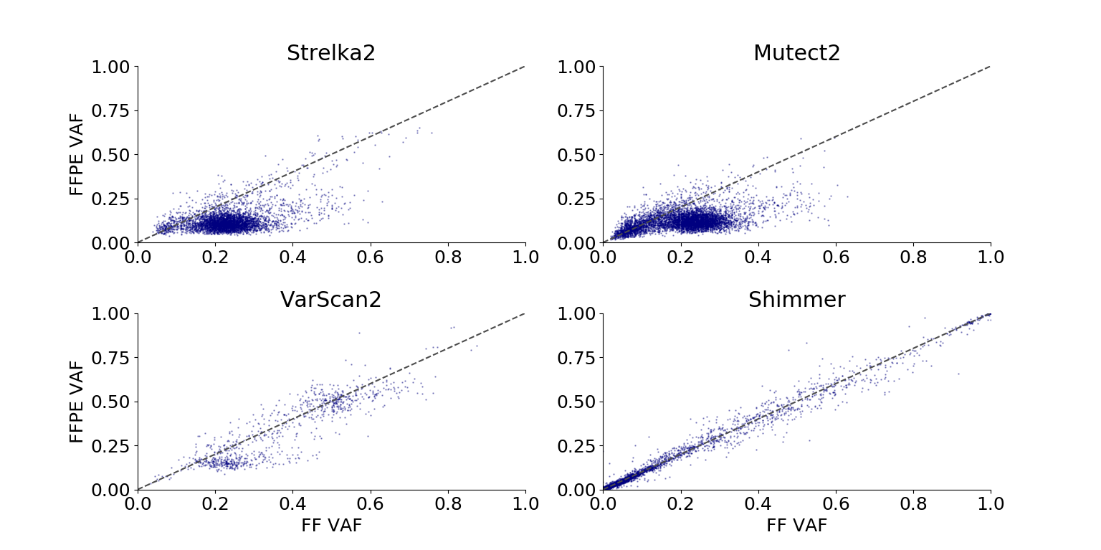


**Supplementary Figure 12.** VAF comparison of calls made in the FFPE and the FF sample per variant caller (UZ001).


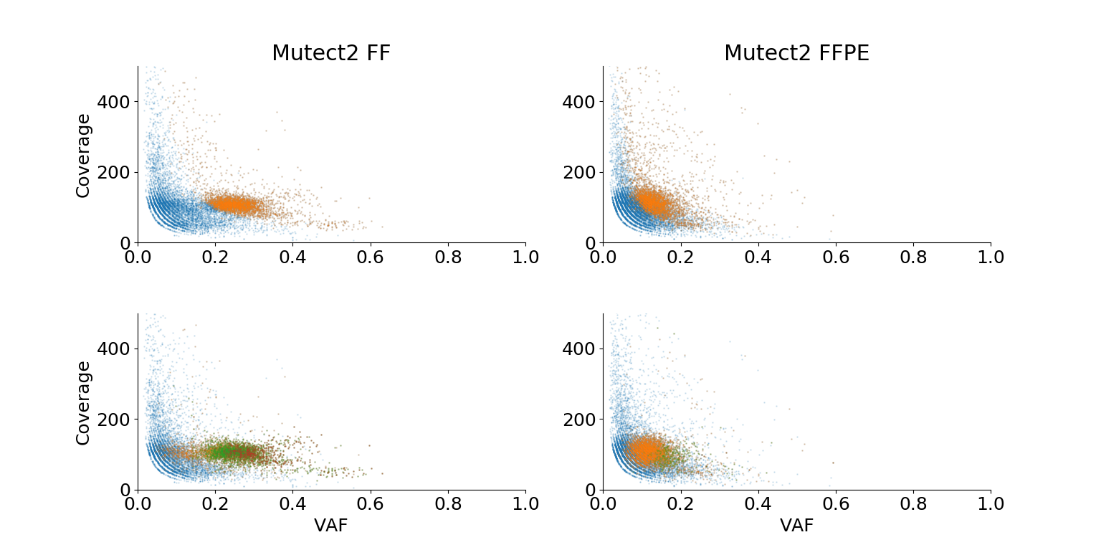


**Supplementary Figure 13.** Coverage versus VAF for variants reported by Mutect2, comparing FF (left) against FFPE (right) (UZ001). The upper panel shows the 25% highest confidence calls in orange and the lower confidence in blue. The lower panel shows which calls are also found by other callers where blue= unique calls, orange = calls reported by 2 callers, green= calls reported by 3 callers, red = calls reported by 4 callers.


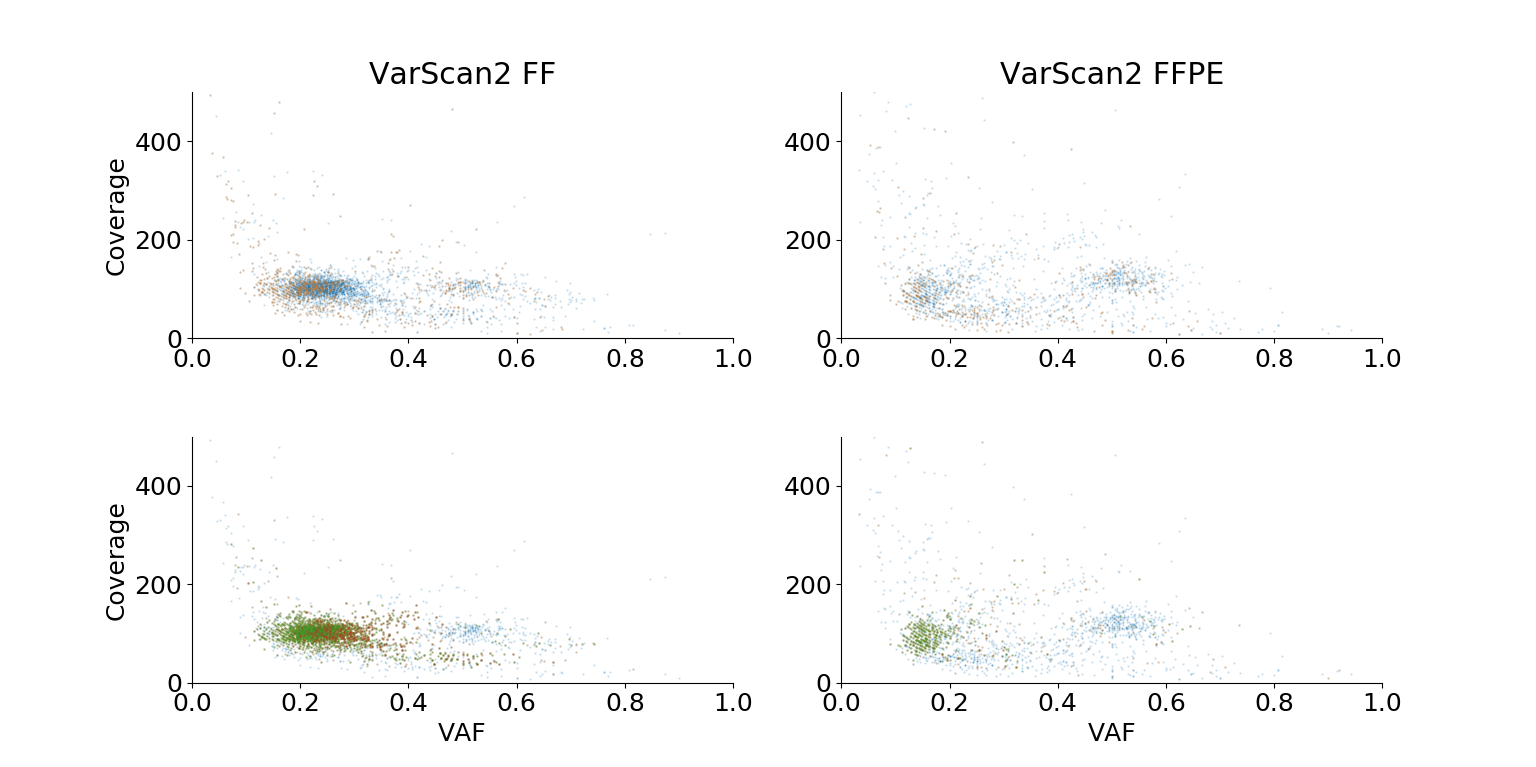


**Supplementary Figure 14.** Coverage versus VAF for variants reported by VarScan2, comparing FF (left) against FFPE (right) (UZ001). The upper panel shows the 25% highest confidence calls in orange and the lower confidence in blue. The lower panel shows which calls are also found by other callers where blue= unique calls, orange = calls reported by 2 callers, green= calls reported by 3 callers, red = calls reported by 4 callers.


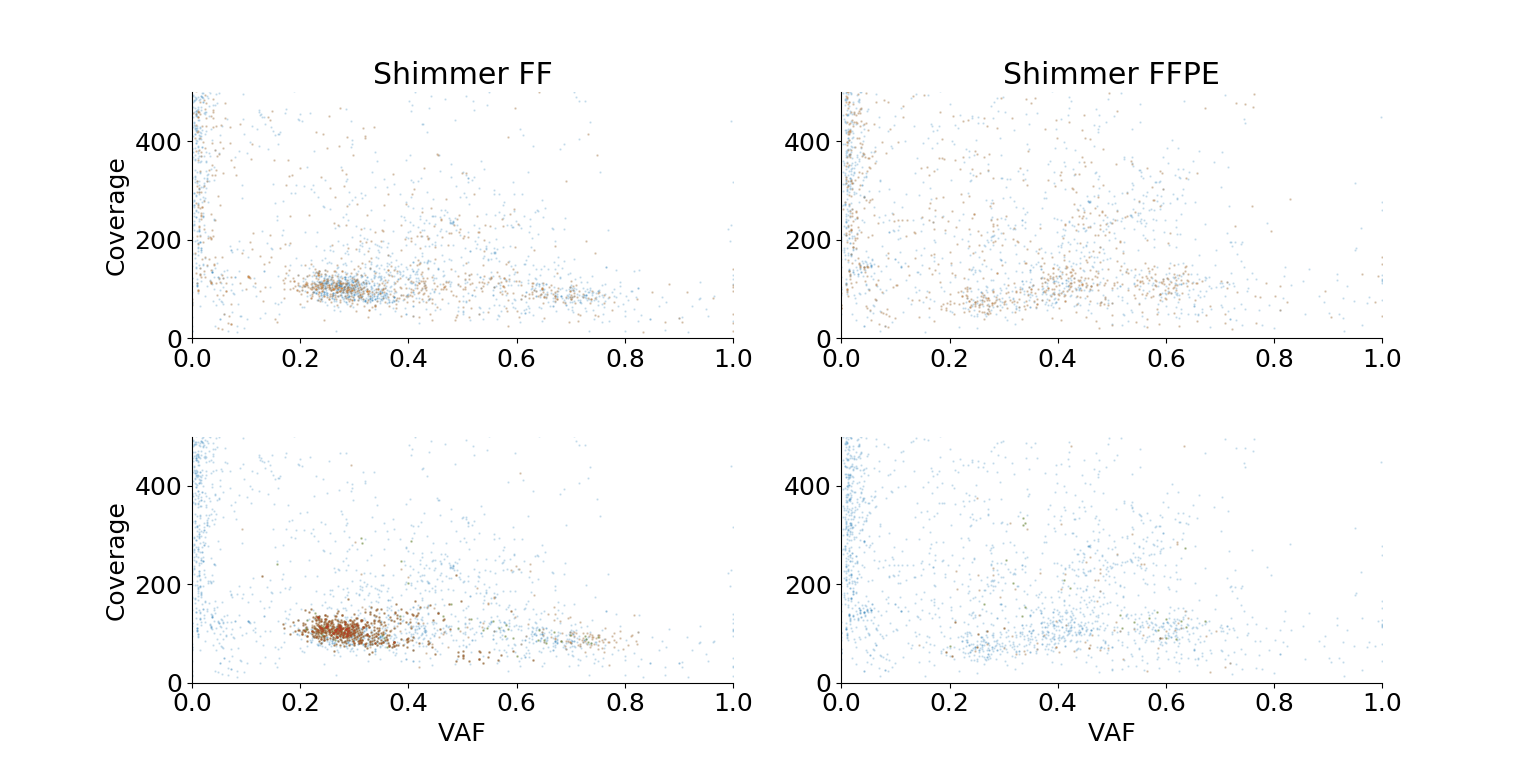


**Supplementary Figure 15.** Coverage versus VAF for variants reported by Shimmer, comparing FF (left) against FFPE (right) (UZ001). The upper panel shows the 25% highest confidence calls in orange and the lower confidence in blue. The lower panel shows which calls are also found by other callers where blue= unique calls, orange = calls reported by 2 callers, green= calls reported by 3 callers, red = calls reported by 4 callers.


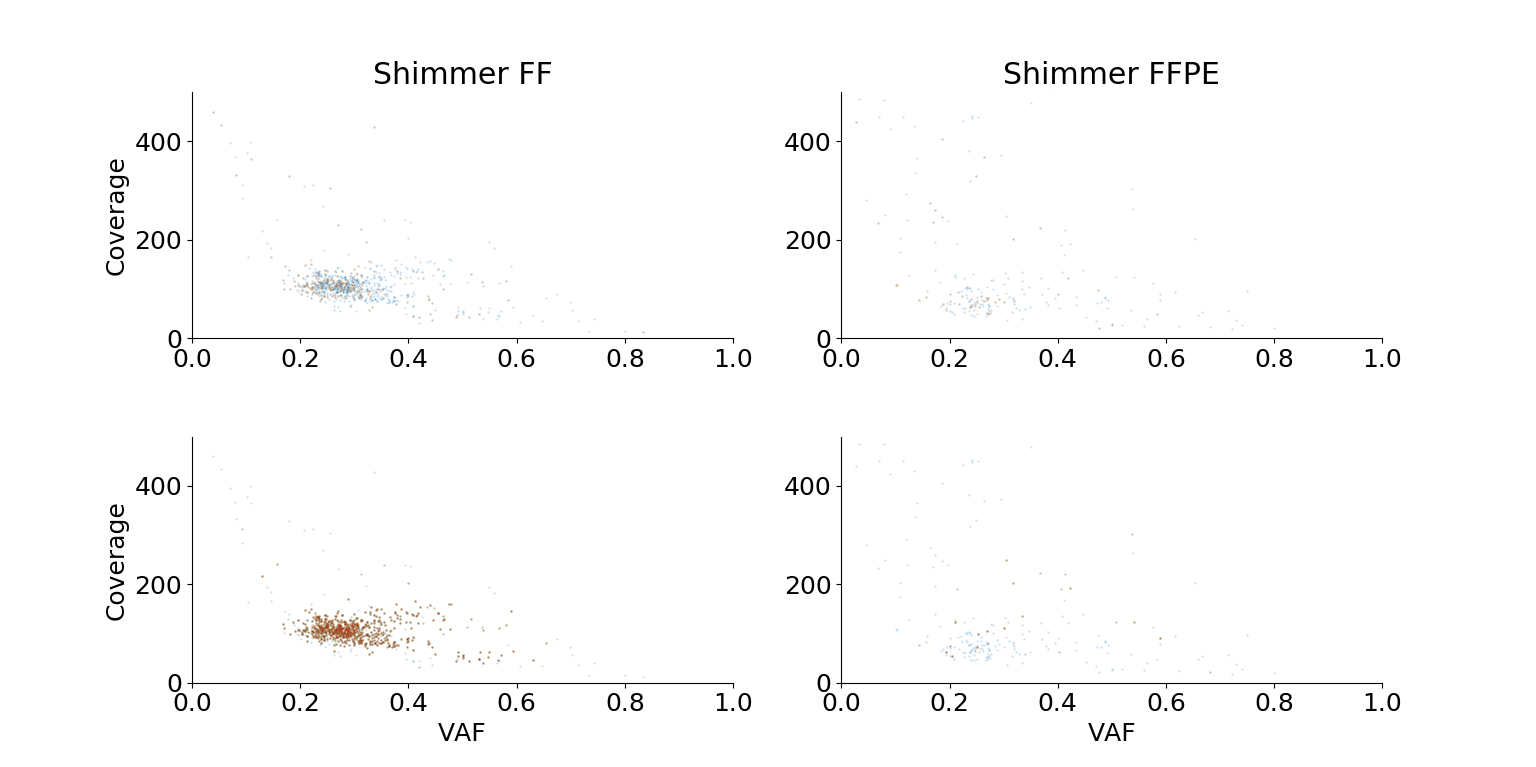


**Supplementary Figure 16.**  Coverage versus VAF for variants reported by Shimmer, comparing FF (left) against FFPE (right) (UZ001). The upper panel shows the 25% highest confidence calls in orange and the lower confidence in blue. The lower panel shows which calls are also found by other callers where blue= unique calls, orange = calls reported by 2 callers, green= calls reported by 3 callers, red = calls reported by 4 callers. In this case, a threshold of VAF = 0 in the normal sample has been imposed.


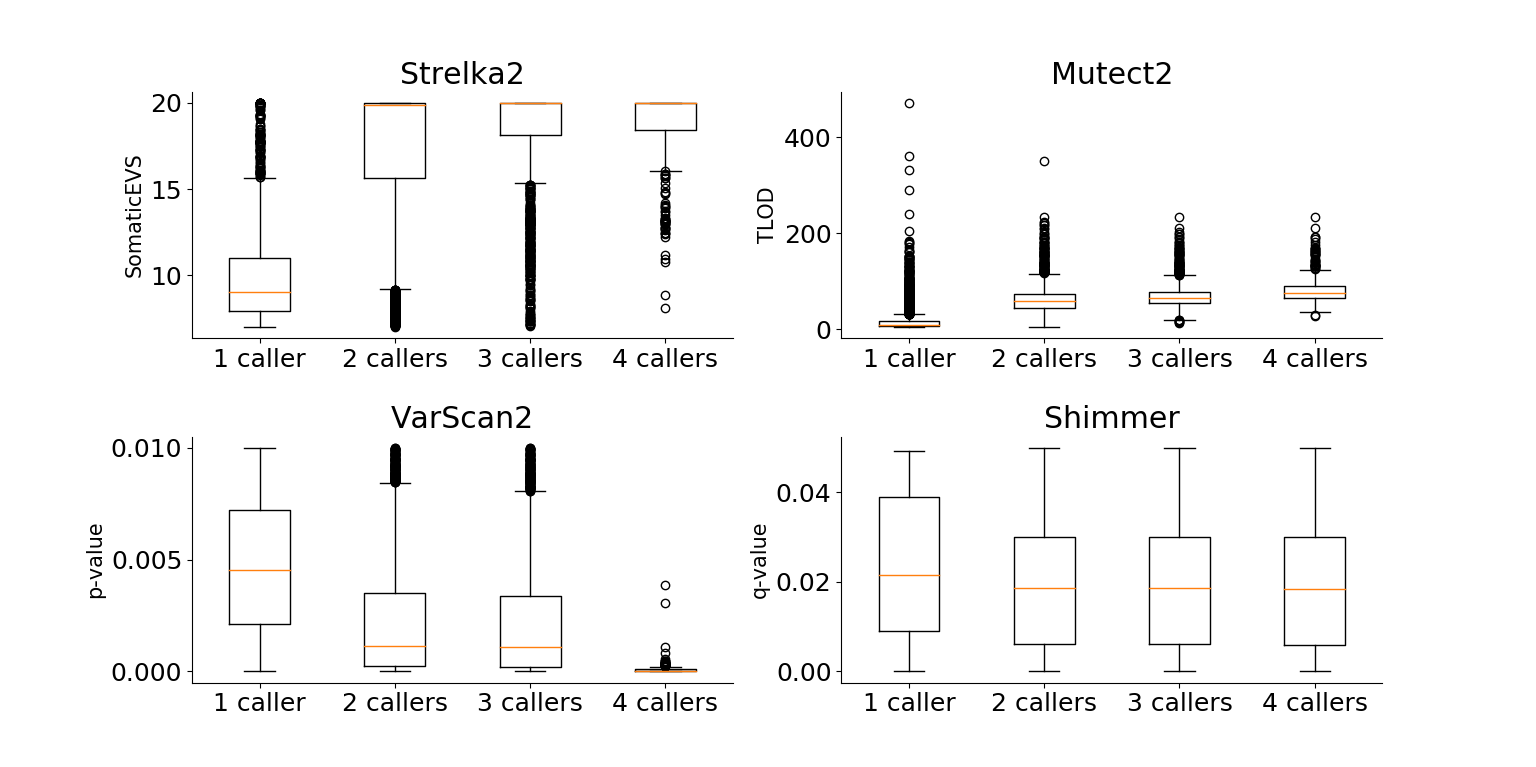


**Supplementary Figure 17.** Boxplots comparing the significance level of calls reported by multiple callers in FF sample (UZ001). Upper left: by Strelka2, upper right: by Mutect2, bottom left: by VarScan2 and bottom right: by shimmer. Median values are displayed in orange.


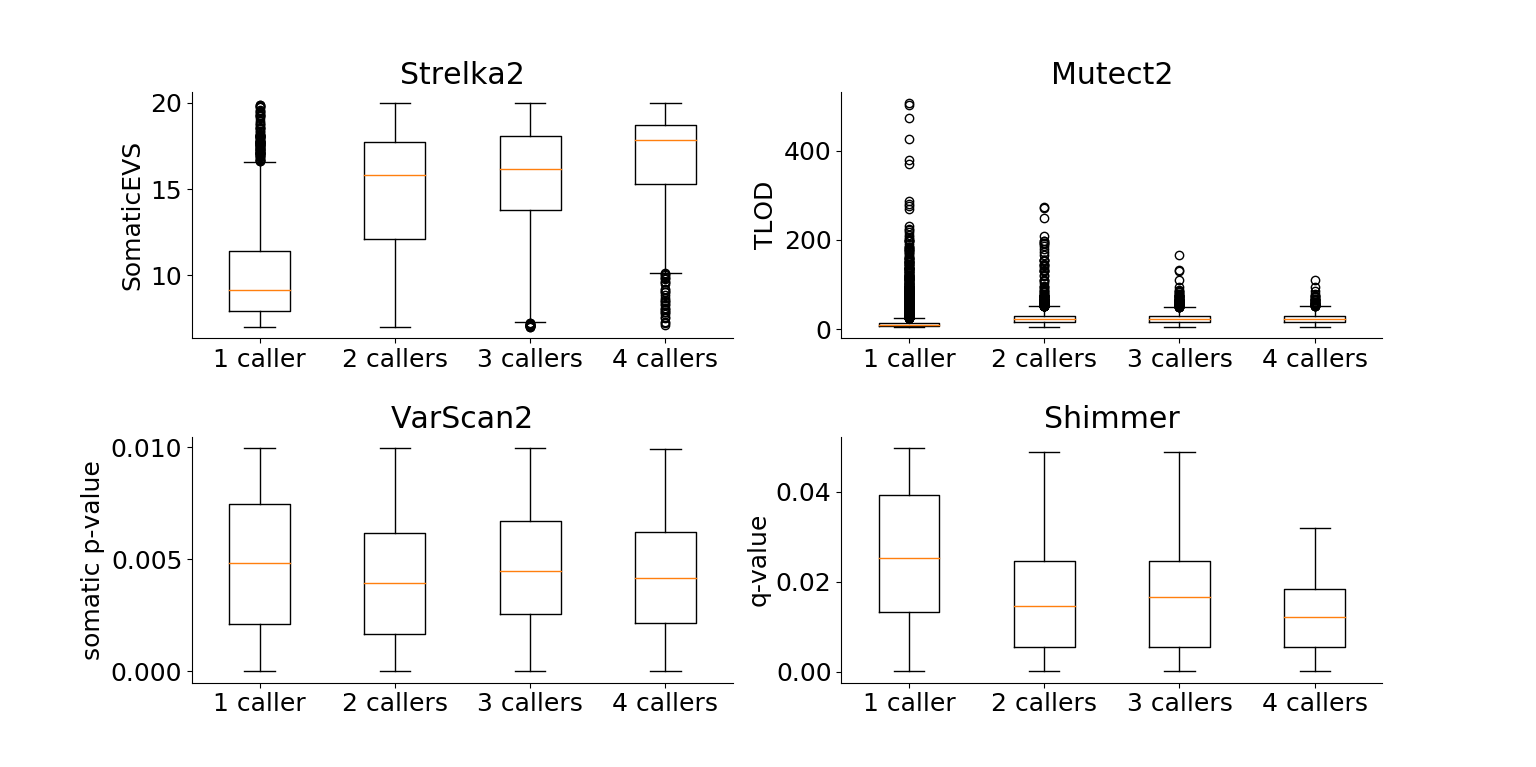


**Supplementary Figure 18.** Boxplots comparing the significance level of calls reported by multiple callers in FFPE sample (UZ001). Upper left: by Strelka2, upper right: by Mutect2, bottom left: by VarScan2 and bottom right: by shimmer. Median values are displayed in orange.


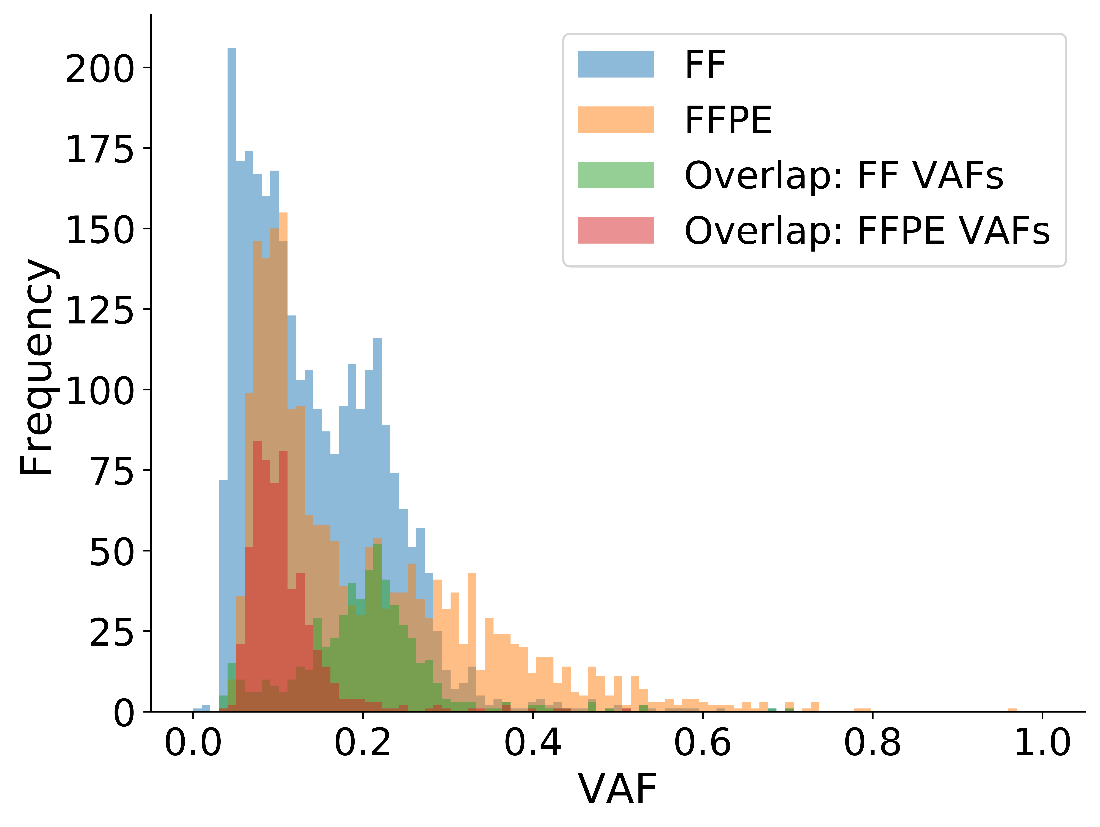


**Supplementary Figure 19.** Distribution of the high confidence variants in FF (blue) and FFPE (orange) (GeL007). The variants present in both FF and FFPE are shown, using their VAFs as calculated in FF (green) and in FFPE (red). Clearly, the common variants have a lower VAF in FFPE, causing them to be classified as subclonal in **Table 7.**
